# Quantifying pluripotency landscape of cell differentiation from scRNA-seq data by continuous birth-death process

**DOI:** 10.1371/journal.pcbi.1007488

**Published:** 2019-11-13

**Authors:** Jifan Shi, Tiejun Li, Luonan Chen, Kazuyuki Aihara

**Affiliations:** 1 Institute of Industrial Science, The University of Tokyo, Tokyo, Japan; 2 LMAM and School of Mathematical Sciences, Peking University, Beijing, China; 3 Key Laboratory of Systems Biology, Center for Excellence in Molecular Cell Science, Institute of Biochemistry and Cell Biology, Chinese Academy of Sciences, Shanghai, China; 4 Center for Excellence in Animal Evolution and Genetics, Chinese Academy of Sciences, Kunming, China; 5 School of Life Science and Technology, ShanghaiTech University, Shanghai, China; 6 Shanghai Research Center for Brain Science and Brain-Inspired Intelligence, Shanghai, China; 7 International Research Center for Neurointelligence, The University of Tokyo Institutes for Advanced Study, The University of Tokyo, Tokyo, Japan; University College London, UNITED KINGDOM

## Abstract

Modeling cell differentiation from omics data is an essential problem in systems biology research. Although many algorithms have been established to analyze scRNA-seq data, approaches to infer the pseudo-time of cells or quantify their potency have not yet been satisfactorily solved. Here, we propose the Landscape of Differentiation Dynamics (LDD) method, which calculates cell potentials and constructs their differentiation landscape by a continuous birth-death process from scRNA-seq data. From the viewpoint of stochastic dynamics, we exploited the features of the differentiation process and quantified the differentiation landscape based on the source-sink diffusion process. In comparison with other scRNA-seq methods in seven benchmark datasets, we found that LDD could accurately and efficiently build the evolution tree of cells with pseudo-time, in particular quantifying their differentiation landscape in terms of potency. This study provides not only a computational tool to quantify cell potency or the Waddington potential landscape based on scRNA-seq data, but also novel insights to understand the cell differentiation process from a dynamic perspective.

## Author summary

Quantifying the Waddington landscape of cell differentiation from high throughput data is a challenging problem in systems biology and biophysics. Here, we propose a theoretical method named LDD (Landscape of Differentiation Dynamics), which builds cell potentials and constructs their differentiation landscape by a continuous birth-death process from scRNA-seq data. This method well exploits the dynamical features of the differentiation process, thus quantifying the differentiation landscape in an accurate manner. We show that LDD can accurately and efficiently build the evolution tree of cells with pseudo-time, in particular quantifying their differentiation landscape in terms of potency. Taken together, this study provides not only a computational tool to quantify cell potency based on scRNA-seq data, but also a theoretical approach to understand the cell differentiation process from a dynamic perspective.

This is a *PLOS Computational Biology* Methods paper.

## Introduction

Single-cell RNA sequencing (scRNA-seq) has become a rapidly developing technique since 2009 when Tang *et al*. [1–3] first proposed the sequencing method. SMART-seq2 [4], CELL-seq [5], Drop-seq [6], and 10X genomics [7] are the most popular protocols at present. They can measure gene expressions for individual cells rather than tissue-level bulk cells without high costs. By analyzing scRNA-seq data, we can determine tissue heterogeneity and capture various developing stages of cells.

Cell differentiation is a process in which several kinds of functional cells arise from one cell type called the pluripotent cell. It is considered that cell specification results from changes in gene expression patterns. As the expression data could be extracted from single cells, many mathematical models have been built, and statistical analysis has been applied to describe the differentiation process, such as RNA velocity [8] and pseudo-time. Pseudo-time is one of the most popular approaches, which attaches a number to each sample or cell as the evolution time from the pluripotent cell. There are mainly two approaches to estimate the pseudo-time of each cell. One is the distance-based method, including Wanderlust/Wishbone [9, 10], Diffusion maps/destiny [11–13], Monocle/Monocle2 [14, 15], scEpath [16] and others. This type of method defines the pseudo-time as the distance from a root cell based on a graph structure. The other type is the entropy-based method, including StemID [17], SLICE [18], SCENT [19], and Markov-chain entropy [20]. This type of method computes some predefined entropy of a cell-cell graph or a gene interaction network as the pseudo-time. We can refer to many comprehensive reviews [21–23] and comparison papers [24–26] for a survey of those works. Although many algorithms have been developed to analyze scRNA-seq data, how to accurately infer the pseudo-time of cells or quantify their potency has not yet been satisfactorily solved. In particular, most of the existing methods are based on statistical measures depending heavily on the samples, or based on the approximation of an equilibrium process, without a dynamical description which is essential for elucidating the differentiation process.

From a modeling viewpoint, cell differentiation is not an equilibrium, but a non-equilibrium, process due to frequent birth and death of cells, and thus can be well modeled by a continuous birth-death (or source-sink diffusion) process. In this paper, derived from such a dynamic process, we propose a new method named Landscape of Differentiation Dynamics (LDD) to analyze the cell differentiation process. Further, we use LDD to compute both the pseudo-time and directed differentiation paths, which are also known as the differentiation landscape. LDD not only quantifies the potency of cells, but also determines the pseudo-time derived from the continuous birth-death process, rather than from the geometric graphical distance used widely in traditional methods. Our method is based on the source-sink diffusion process, which exploits the dynamical features of the stochastic differentiation process, thus quantifying the differentiation landscape in an accurate manner from a dynamic perspective.

In this study, we constructed the potential landscape *V*(***x***) by solving Eq (3) under the non-equilibrium steady state assumption of the differentiation dynamics. One key observation of our work was that we could obtain the net-flow rate *R*(***x***) from the data without assuming its prior knowledge, which advances previous proposals on non-equilibrium dynamics of gene expression [27]. LDD is a two-level algorithm: one at the single cell level, which quantifies cell heterogeneity for each cell based on the diffusion process, and the other at the cluster level, which quantifies the transition between cell types (or clusters) based on the Markov process. Additionally, the reverse of the pseudo-time could be calculated for each cell type. Therefore, cells with the highest potential are deemed pluripotent and will evolve into differentiated cells with lower potential. Lineages or differentiation branches could be detected from a transition matrix between different clusters. From the differentiation landscape constructed by LDD, we could clarify the global landscape structure of a real biological process. Further, pluripotent cells with higher potential in our study were quantitatively shown to differentiate into downstream cells with lower potential by LDD, similar to a ball rolling down a mountain as described by the Waddington landscape [28]. Taken together, this study provides not only a new dynamical model with a computational tool to quantify the cell potency based on scRNA-seq data, but also a new approach to understand the differentiation process from a dynamic and stochastic perspective.

## Results

### Modeling cell differentiation by continuous birth-death process

Cell differentiation is clearly a non-equilibrium process due to frequent birth and death of cells. Thus, to model the cell differentiation, we use the continuous birth-death process as the underlying dynamics of cell differentiation, which is also named as the source-sink Fokker-Planck equation in mathematics and the population balance equation in Klein *at al*.’s work [27, 29, 30]. The continuous birth-death process assumes that the probability density function *c*(***x***, *t*) of all sample cells develops as

$$\begin{array}{c} \frac{\partial c(x,t)}{\partial t}=\nabla \cdot \left(c\left(x,t\right)\nabla F\left(x\right)\right)+D\Delta c\left(x,t\right)+R\left(x\right)c\left(x,t\right), \end{array}$$ (1)

where ***x*** is a vector of gene expression, *t* is the time, *F*(***x***) is a potential function, *D* is the noise amplitude, and *R*(***x***) is the net-flow of cells at state ***x***. ∇, ∇⋅, and Δ denote the gradient, divergence, and Laplace operators, respectively. When the system reaches a non-equilibrium steady state, i.e. lim_*t*→∞_ *c*(***x***, *t*) = *p*(***x***) or ∂*c*(***x***, *t*)/∂*t* = 0, the potential can be decomposed as *F*(***x***) = *U*(***x***) + *V*(***x***) and calculated by

$$\begin{array}{c} U(x)=-D\log p(x), \end{array}$$ (2)

$$\begin{array}{c} LV(x)=[\nabla \log p(x)\cdot \nabla +\Delta ]V(x)=-R(x), \end{array}$$ (3)

according to [27]. We name the flow that is differentiation-oriented as “advection”, while we use the term “diffusion” which is caused by random noise [31–33]. *U*(***x***) is known as the equilibrium potential caused by diffusion without birth and death, and *V*(***x***) is a new potential caused only by advection without diffusion. By definition, noise is known to only influence the diffusion process and generates meta-stable wells in *U*(***x***). Therefore, *V*(***x***) can be taken as the cell differentiation potential to describe the differentiation direction. The cells will evolve from pluripotent cells with high *V*(***x***) to differentiated cells with low *V*(***x***). *V*(***x***) can represent the Waddington landscape [28] of the cell at state ***x***, and the additive inverse of *V*(***x***) can be considered as a reflection of the pseudo-time (see Materials and methods).

### Estimating cell potential V(x) and constructing differentiation paths

To obtain *V*(***x***) from Eq (3), the net-flow *R*(***x***) and backward operator L=∇logp(x)·∇+Δ need to be estimated from scRNA-seq data. Due to the limitation of sample size, it is difficult to accurately measure net-flow *R*(***x***) for every cell. In contrast to [27], which required additional information, we first clustered samples into different cell types/clusters, and then computed the net-flow ${\hat{R}}_{s}$ for each cluster *s* from the gene expression matrix by the divergence theorem and marginal decomposition. On the other hand, benefiting from the diffusion map theory [34, 35] and model reduction [36], the backward operator L was approximated by $\hat{L}$, which was the coarse-grained discrete matrix representation of L between cell clusters, obtained from the cell-to-cell transition matrix. Thus, with the approximated net-flow $\hat{R}$ for every cluster/cell type, and the approximated transition operator $\hat{L}$, $\hat{V}$ could be obtained numerically, generating a concrete value for the potency of each cell type. Additional details can be found in Materials and methods. We remark that in [27], the net-flow rate was set as the prior knowledge for each cell. However, we were able to obtain the value from the gene expression matrix if cells were clustered into different metastable states. This is one key point of our work.

**Fig 1:**
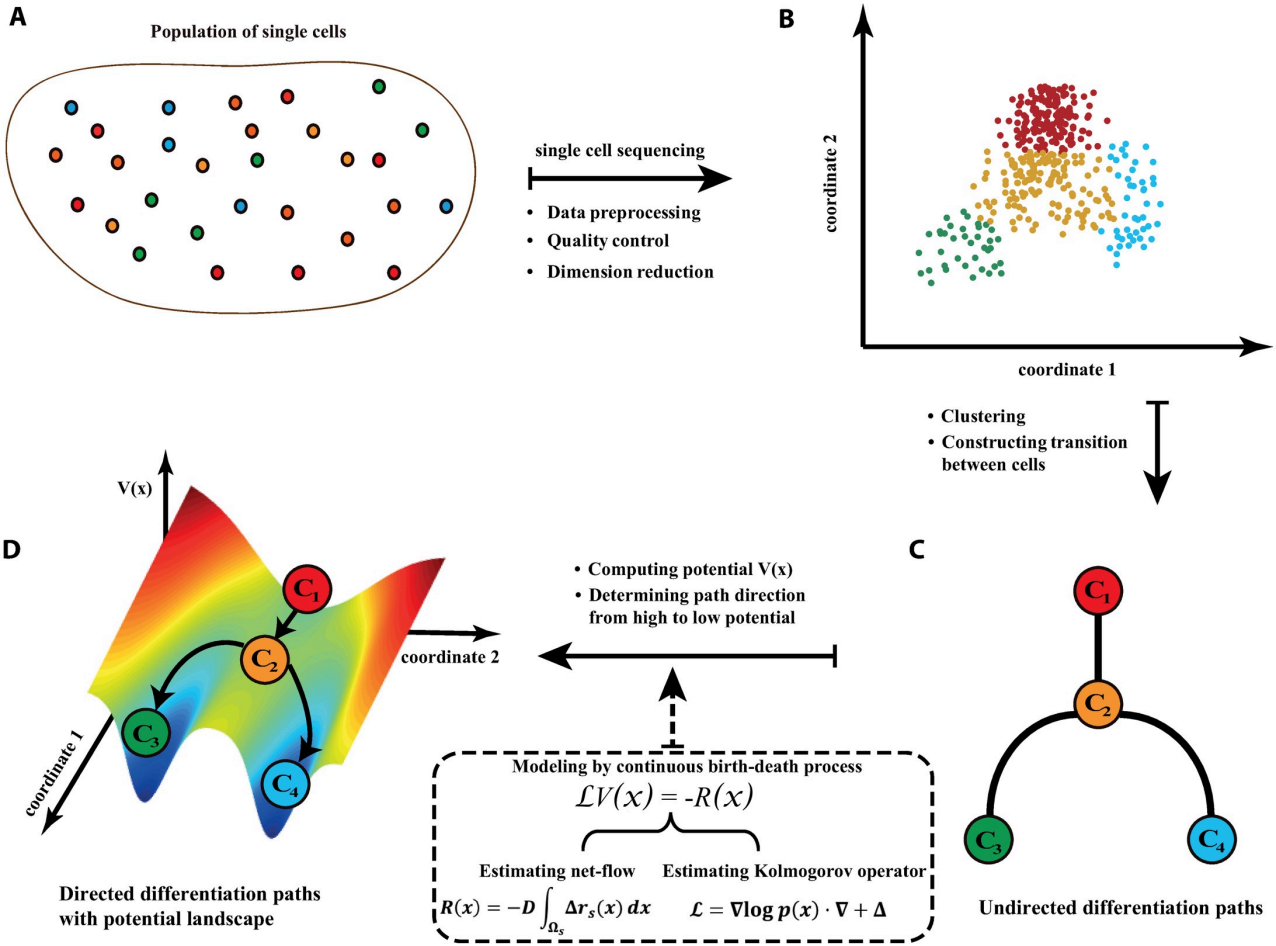
Flowchart of the Landscape of Differentiation Dynamics (LDD) method. **A**: A pool of single cells, from which we can obtain the gene expression matrix by single cell sequencing. **B**: After preprocessing, quality control, and dimension reduction, a low-dimensional data matrix is obtained. **C**: The samples are clustered into different types. Undirected differentiation paths are determined by a transition matrix between clusters. **D**: After applying the continuous birth-death process to model the whole differentiation process, the potential *V*(***x***), differentiation directions, and landscape can be constructed.

To illustrate the entire differentiation process, we constructed its landscape, in which nodes were cell types with potential $\hat{V}$, paths were determined by the transition matrix between clusters, and directions were from high to low potential. We named this procedure as Landscape of Differentiation Dynamics (LDD). Fig 1 provides a flowchart of LDD, which is described in details in Materials and methods. An algorithmic description is provided in S1 Text section S1 and Fig I in S1 Text.

### Computing differentiation landscape of simulated models

**Fig 2:**
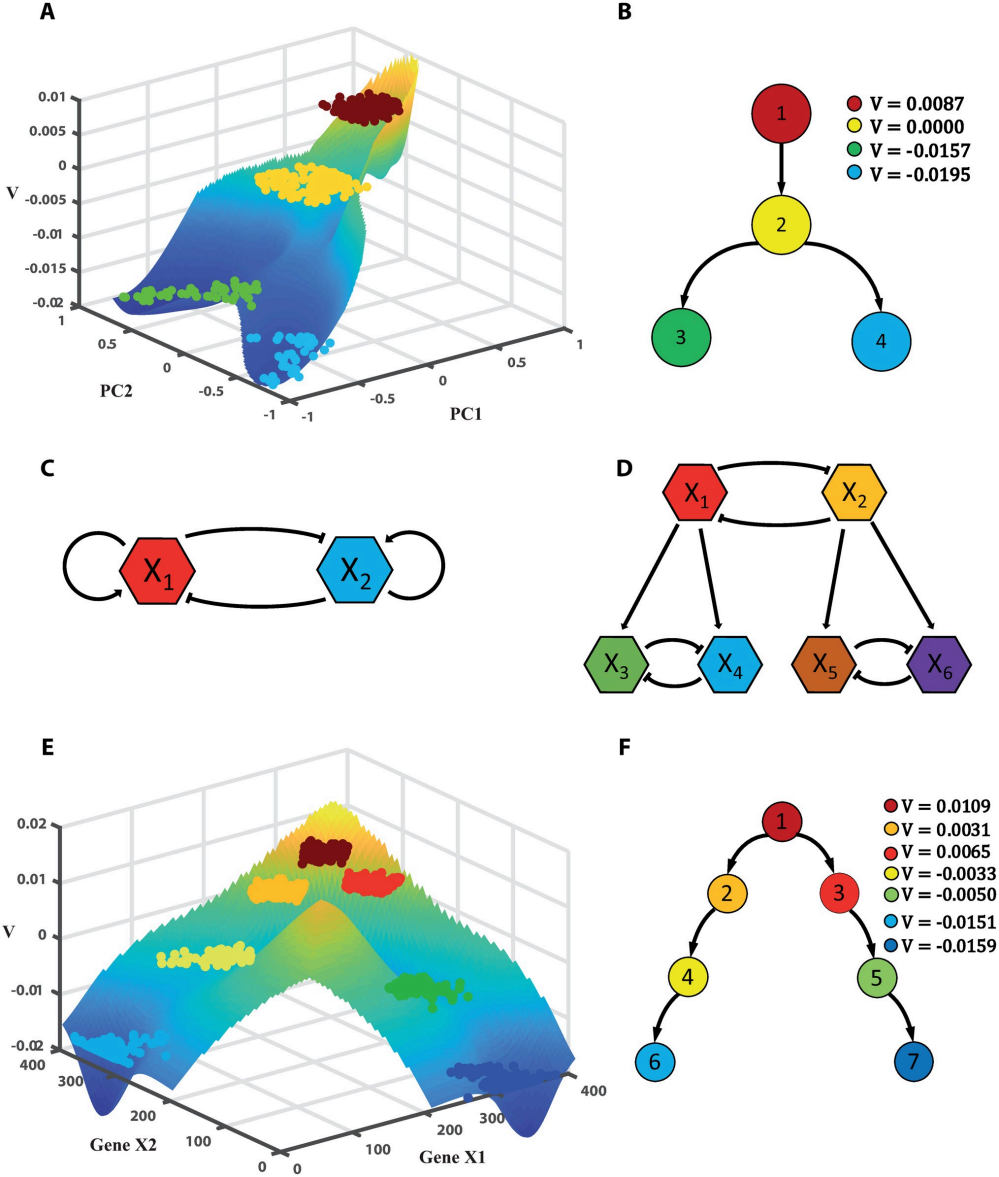
Differentiation landscape, differentiation paths, and gene networks for simulated models. **A** and **B** are the LDD potential landscape and differentiation paths using data from the simulated drift-diffusion process, in which samples/cells were clustered into four groups. **C** and **D** are the two-gene and six-gene regulatory networks for simulation, respectively. For the two-gene network, the potential landscape and differentiation paths for clusters are shown in **E** and **F**, respectively, where seven clusters were detected. Constant potential *V* for each cluster was computed by LDD, while the landscape is an illustration constructed by the method in Materials and methods.

We used three simulated models to construct potential and verify our LDD method. The first is a drift-diffusion process, in which particles evolve by

$$\begin{array}{c} x\left(t+\Delta t\right)=x\left(t\right)-\nabla F\left(x\left(t\right)\right)\cdot \Delta t+\sqrt{D\Delta t}\cdot \xi \left(t\right), \end{array}$$ (4)

where ***x*** is the position of a particle in 50 dimensions, *F* is a potential function, *D* is the noise amplitude, and ***ξ*** is a normal random vector standing for noise. A bifurcation from one branch to two branches occurred in the system, representing cell differentiation. Particles/cells were generated from a source, and two sinks indicated places for particle/cell removal or death. It imitated the cell’s lifespan from birth to death. Details about the model are shown in S1 Text. In the example, 400 samples/cells were simulated. After reducing to two dimensions using principal component analysis (PCA) and clustering the 400 samples into four groups/clusters by k-means, we computed the potential $\hat{V}$ of each cluster. The landscape in a three-dimensional view is illustrated in Fig 2A. Fig A(a) in S1 Text shows the potential in a two-dimensional space. In Fig 2B, the differentiation paths between the four clusters were constructed. The cluster with the highest potential corresponded to the source region, and the two sinks with low potential values were at the end of two lineages.

The next two simulated examples are two-gene and six-gene regulatory networks. Their gene interactions are shown in Fig 2C and 2D, respectively. A region for cell birth is shown, as well as several regions for cell death. The detailed simulation method can be found in S1 Text. For the two-gene network, as the genes inhibit each other, two lineages formed. In each branch, one gene had high expression, while expression of the other gene was very low. The illustrative landscape for the two-gene network is in Fig 2E, and the two-dimensional plot is in Fig A(b) in S1 Text. Fig 2F shows the differentiation paths between seven groups. LDD generated the potential in a correct order, i.e. pluripotent cells with higher values, and differentiated cells with lower values. Similar results of LDD were obtained in the six-gene network, which had four branches for the samples. Only two genes had high expression, while the others were low in each branch, which are shown in Fig A(c) and A(d) in S1 Text.

The three examples demonstrate the ability of LDD to quantitatively characterize the stochastic process of cell differentiation from a dynamic perspective, due to our model based on the continuous birth-death process. In particular, only using the observed data, LDD could quantitatively show that pluripotent cells with higher potential differentiated into downstream cells with lower potential, similar to a ball rolling down a mountain as described by Waddington [28].

### Computing differentiation landscape of real datasets

To further verify the efficiency of LDD, we applied it to four real datasets, i.e. Guo’s dataset, Nef’s dataset, Xu’s dataset, and Furlan’s dataset.

Guo’s dataset [37] describes cells developing from zygote to blastocyst, through oocyte, 2-cell, 4-cell, 8-cell, morula, E3.5 blastocyst, and E4.25 blastocyst stages. At the end term of morula, the cells could differentiate into trophectoderm (TE) and inner cell mass (ICM). Nef’s dataset [38] focused on the determination of mouse’s sex. 400 samples were selected from E10.5, E11.5, E12.5, E13.5, and E16.5 stages. Two branches named the interstitial progenitor cell lineage and Sertoli cell lineage appeared during the observation time. Xu’s dataset [39] showed that the progenitor hepatoblasts had bipotency to divide into hepatocytes and cholangiocytes. Furlan’s dataset [40] found that a large number of chromaffin cells in adrenal medulla arose from a kind of peripheral glial stem cell called Schwann cell precursors (SCPs). The description and the preprocessing of these datasets can be found in S1 Text.

**Fig 3:**
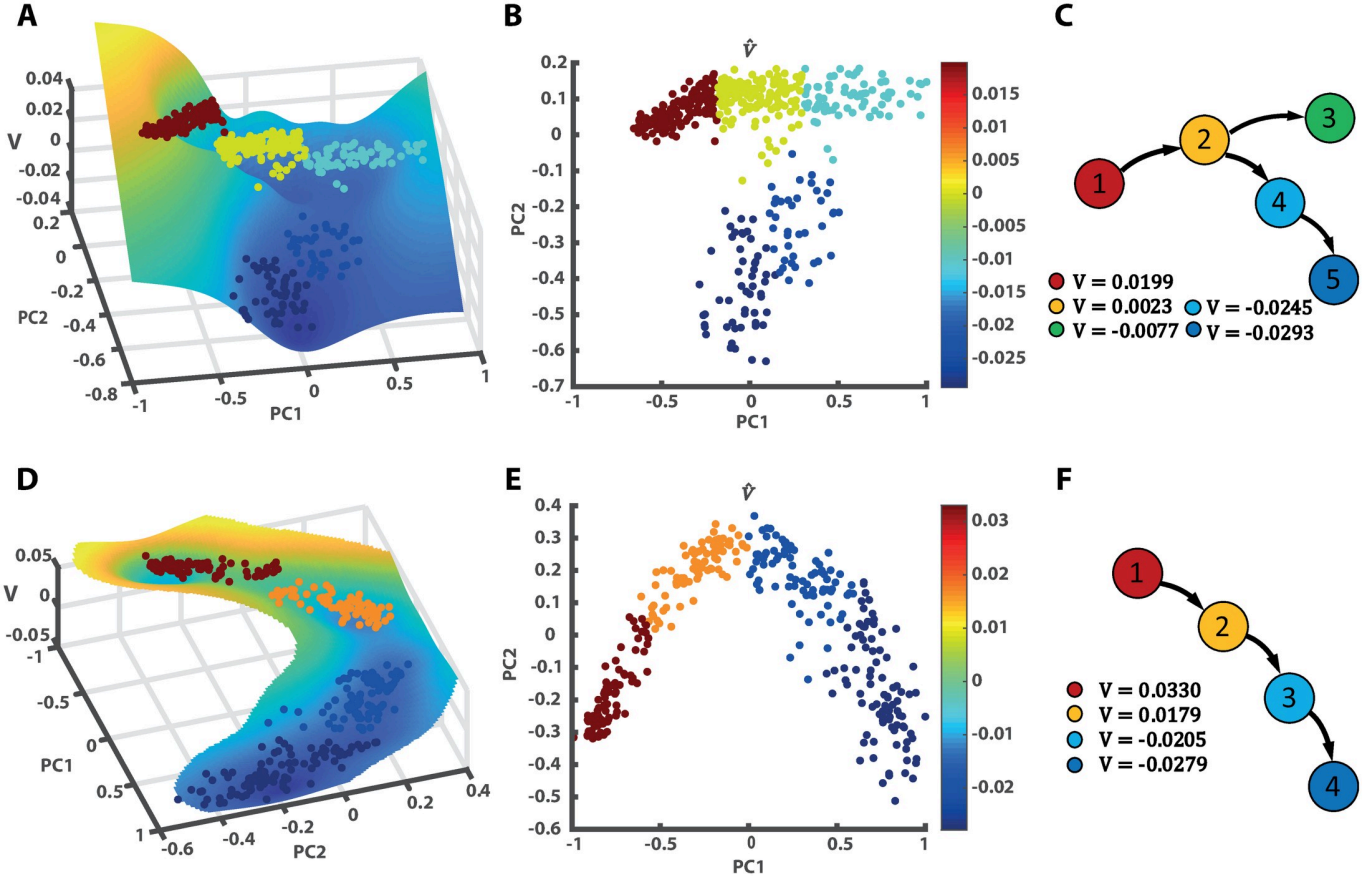
Differentiation landscape and differentiation paths for real datasets. **A**, **D** are the LDD potential landscapes, **B**, **E** are the potential values plotted in the two-dimensional reduction space, and **C**, **F** are the differentiation paths. **A**-**C** use Xu’s dataset, which describes that hepatoblasts (cluster 1) differentiate into hepatocytes (cluster 3) and cholangiocytes (cluster 5). **D**-**F** use Furlan’s dataset and show that chromaffin cells (cluster 4) are generated from SCPs (cluster 1).

Using LDD, we computed the potential and differentiation paths of each dataset. The illustrative landscapes, potential values, and lineages of Xu’s dataset are displayed in Fig 3A–3C, which show that the progenitor hepatoblasts (cluster 1) with high potency differentiated into hepatocytes (cluster 3) and cholangiocytes (cluster 5) with low potency. Fig 3D–3F are the corresponding results of Furlan’s dataset. There was a single path through which the SCPs became chromaffin cells. For Guo’s dataset and Nef’s dataset, the results are given in Fig B in S1 Text. For Nef’s dataset, we also computed Pearson correlations between LDD potential and three character genes *Pbx1*, *Gpc3*, and *Sfrp1* as 0.9015, 0.8582, and 0.8000. These genes decreased during the differentiation process, which indicated that the pseudo-time conformed to the differentiation direction.

### Comparison with other pseudo-time algorithms

From both the simulated and real datasets, we showed that LDD could obtain the correct differentiation paths by quantifying potential values of cells. The pseudo-time is another important topic in single cell research, which provides a time label to each cell. We set the additive inverse of potential $\hat{V}$ as our pseudo-time by LDD, and compare it with several traditional methods. Because entropy-based methods usually need additional information, such as gene interactions or function annotations, we only compared LDD with six distance-based methods, i.e. TSCAN [41], Monocle2 [15], Diffusion Map [11, 12], DPT [13], SLICER [42], and Slingshot [43]. Their properties are listed in Table 1, and the details are given in S1 Text.

**Table 1:** Properties of different pseudo-time methods.

	LDD	TSCAN	Monocle2	Diffusion map	DPT	SLICER	Slingshot
Basic Model	Birth-death Dynamics	Distance on tree/graph	Distance on tree/graph	Distance on tree/graph	Distance on tree/graph	Distance on tree/graph	Distance on tree/graph
Not use a root cell as input	Yes	No	No	No	No	No	No
Dimension Reduction	Yes	Yes	Yes	Yes	Yes	Yes	Yes
Clustering	Yes	Yes	No	No	No	No	Yes
Detecting ⩾ 1 branches	Yes	Yes	Yes	Yes	Yes	Yes	Yes
Reference	—	[41]	[15]	[11, 12]	[13]	[42]	[43]

Each column is a method. For the rows, “Basic Model” indicates the theoretical model used in the method. “Not use a root cell as input” indicates whether the algorithm requires a start cell as input. For some algorithms if giving a wrong root cell, the pseudo-time could be reverse of the true time direction or even be totally messed. “Dimension reduction” indicates whether dimensional reduction is applied on the data matrix. “Clustering” states whether the algorithm clusters cells during the process. “Detecting ⩾ 1 branches” indicates whether the algorithm could find more than one different branches/lineages. The corresponding papers are listed in the “Reference” row.

For the simulated datasets, we chose the Pearson correlation between the pseudo-times and the true-time labels as the measurement. The second to the fourth columns in Table 2 show the results for the Simu1 (the drift-diffusion process), Simu2 (the two-gene regulatory network), and Simu3 (the six-gene regulatory network) datasets. The closer the Pearson correlation approximated to 1, the more reliable the algorithm was. LDD and DPT outperformed the others; however, DPT required additional information, i.e. a started root sample to represent the stem cell. Hence, it implies that LDD effectively uses the information in the samples, which leads to correct differentiation paths.

**Table 2:** Comparison between seven pseudo-time methods.

	Pearson Correlation *ρ*			Wilcoxon Ranksum Test p-value			
Dataset	Simu1	Simu2	Simu3	Guo	Nef	Xu	Furlan
Measure	Correlation *ρ* between pseudo-time and true time			Oocyte < E4.25 blastocyst	E10.5< E16.5	E10.5 < E17.5	SCPs < chromaffin
LDD	**0.9135**	0.9520	0.8229	**3.842e-09**	9.432e-20	**3.557e-24**	**2.104e-39**
TSCAN	0.1343	0.0404	0.0055	1.344e-01	2.588e-01	6.145e-16	2.067e-34
Monocle2	0.0668	0.0121	0.0131	2.370e-07	3.084e-01	8.479e-22	2.067e-34
Diffusion map	0.7707	0.0398	0.0069	2.370e-07	6.277e-09	8.479e-22	2.067e-34
DPT	0.9079	**0.9603**	**0.8797**	2.370e-07	1.053e-15	8.479e-22	2.067e-34
SLICER	0.7348	0.8807	0.4514	3.672e-07	**4.921e-22**	8.479e-22	2.067e-34
Slingshot	0.0971	0.0147	0.0866	2.370e-07	3.725e-19	6.781e-22	2.067e-34

Seven pseudo-time methods, including LDD, were tested on three simulated datasets and four real datasets. For the simulated datasets, as we had true time labels for every cell, the Pearson correlation between true times and pseudo-times was calculated. For real datasets, we used the one-sided Wilcoxon ranksum test to determine whether pluripotent cells had earlier pseudo-time than differentiated cells. The alternative hypothesis is listed in the third row in the table. Bold numbers are the best method in its column. LDD performs among the best, while the other six methods require a root cell as prior information.

For the real datasets, the accurate cell time is unavailable; however, several stage-level time labels, such as E10.5 and E11.5, are given. Under one stage label, there are several cell types. Therefore, instead of the Pearson correlation, we used the one-sided Wilcoxon ranksum test to determine whether the stem cell stage had earlier pseudo-time than the differentiated stage. The p-value was chosen as the measurement [19, 20]. The fifth to the eighth columns in Table 2 list the p-values for the four real datasets (Guo’s, Nef’s, Xu’s, and Furlan’s). The alternative hypothesis was that pluripotent stem cells had earlier pseudo-time than differentiated cells. Thus, the smaller the p-value was, the better the algorithm performed. From Table 2, LDD was always shown to produce good results among the seven methods (the second place in Nef’s dataset and the best in other three datasets).

The conclusion from the comparison between different pseudo-time methods is as follows: LDD and DPT performed well in determining the pseudo-time but DPT required additional information, i.e. a root cell. Thus, LDD is an efficient approach to model cell differentiation and quantitatively characterize the Waddington potential landscape or differentiation paths by exploiting dynamical and stochastic features of the differentiation process from the measured data.

## Discussion

In this paper, in contrast to the approximation of the equilibrium process widely used in previous methods, we used a stochastic non-equilibrium steady process, i.e. a continuous birth-death process model, to describe the differentiation dynamics of cells, which well captures the dynamical and stochastic features of the cell differentiation process. LDD based on this model was proposed to compute the cell’s potency value and construct the differentiation paths/landscape from scRNA-seq data, derived from the diffusion map theory and divergence theorem. Using the landscape, we showed that cells developed from high to low potential. Different lineages were also detected by the transition matrix obtained by LDD. As the LDD’s pseudo-time (the additive inverse of potential) originates from a dynamic system, it gets rid of the limitation of distance measures on graphs. Comparison studies with traditional methods also showed that it was a powerful and effective method on both simulated and real datasets.

There are still some features and issues to be discussed: (1) The dynamical model used in LDD requires that the sample cells are in a non-equilibrium steady state, i.e., the cells’ birth rate should be equal to the death rate. In a short time period when the environment does not change much, the model works, but for a long-term study, this condition may not hold. (2) As the dynamical model describes a continuous flow of differentiation, the sampled cells need to represent the whole differentiation process. When gathering data, this requirement should be considered. (3) Cell clustering is another important issue. Clustering cells in our method requires that different types are separated into different metastable states. However, our samples/cells also need to keep the continuity of differentiation. Thus, there is an appropriate balance between separability and continuity. In our examples, PCA and k-means clustering can maintain the orthogonal invariance for the landscape, which may not occur with other nonlinear approaches. Therefore, the method must be chosen carefully to ensure the system’s properties (see Materials and methods). (4) The requirement on the samples is unclear and ambiguous for the distance-based or entropy-based methods, which limits their applications. In contrast, LDD provides a general mechanism for the differentiation process from a stochastically dynamic viewpoint, and its results are guaranteed by the diffusion model for the measured samples. (5) The birth-death process modeling is inspired by [27], but we further improve its theoretical result. In particular, we remove the requirement for measuring net-flow, i.e. we show that net-flow ${\hat{R}}_{s}$ can be directly computed from the gene expression matrix by the divergence theorem and marginal decomposition. In addition, rather than the graph Laplacian, we applied diffusion map theory, which considers the weights on edges and is a generalization of the graph Laplacian. (6) Saelens et al. [26] gave a comprehensive comparison over 45 scRNA packages, in aspects of their accuracy, scalability, stability, and usability. Our study focused on the theoretical framework and algorithm construction. Further improvements and tests of our algorithm on multiple and larger datasets will be performed as our future work.

In summary, by the diffusion map theory and divergence theorem, we provide a new approach to quantify cell differentiation from measured scRNA-seq data based on the continuous birth-death process, which well exploits the dynamical features of the cell differentiation process from a dynamic landscape viewpoint. The essential evolution laws of cells need much more efforts through joint experimental and theoretical studies in the future.

## Materials and methods

### Cell differentiation dynamics as continuous birth-death process

We use a stochastic non-equilibrium process Eq (1) to model the cell differentiation, which is also named the source-sink Fokker-Planck equation or the population balance equation [27, 29, 30]. In Eq (1), the function *c*(***x***, *t*) represents the probability density function (pdf) of cells at ***x***, which will be estimated from scRNA-seq data. The term *R*(***x***) is crucial to understand the birth and death of the cells involved in the system. If there is a source and cells are increasing locally, *R*(***x***) will be positive. Conversely, if there is a sink and cells are removed or die, *R*(***x***) will be negative.

### Deriving cell differentiation potential V(x)

Assume that the system reaches a non-equilibrium steady state, which means cells keep being born and dying but the whole population distribution is invariant. Let *t* → ∞, then we will obtain from Eq (1)

$$\begin{array}{c} \nabla \cdot (p(x)\nabla F(x))+D\Delta p(x)+R(x)p(x)=0, \end{array}$$ (5)

where *p*(***x***) = lim_*t*→∞_ *c*(***x***, *t*). Only depending on *p*(***x***), *F*(***x***) could have many solutions, one of which can be written as

$$\begin{array}{c} \nabla \cdot (p(x)\nabla U(x))+D\Delta p(x)=0, \end{array}$$ (6)

$$\begin{array}{c} \nabla \cdot (p(x)\nabla V(x))+R(x)p(x)=0, \end{array}$$ (7)

and

$$\begin{array}{c} F(x)=U(x)+V(x). \end{array}$$ (8)

The explicit forms for Eqs (6) and (7) are

$$\begin{array}{c} U(x)=-D\log p(x), \end{array}$$ (9)

$$\begin{array}{c} LV(x)=-R(x), \end{array}$$ (10)

where L is the backward Kolmogorov operator

$$\begin{array}{c} L=\nabla \log p(x)\cdot \nabla +\Delta . \end{array}$$ (11)

*U*(***x***) can be considered as the equilibrium potential caused by diffusion without samples’ birth and death, while *V*(***x***) can be taken as the potential caused by a birth-death flow without diffusion. If the noise amplitude *D* approaches zero, the diffusion vanishes and thus we can take *V*(***x***) as the potential to describe cell pluripotency, or as the reverse pseudo-time of cell differentiation.

To compute *V*(***x***), instead of setting *R*(***x***) as the given values at each point ***x*** like [27], we cluster samples into groups/types to obtain $\hat{R}$ for each group only from the expression matrix. The backward operator L is also approximated by a coarse-grained $\hat{L}$ defined on the cell clusters. The cell differentiation potential $\hat{V}$ is then computed for each cluster based on a discrete version of Eq (10). The following subsections will show both theoretical and numerical details, and the overall algorithm is described in S1 Text.

### Constructing Kolmogorov operator L by diffusion map from cell samples

To approximate the backward Kolmogorov operator L in Eq (11), we utilize the diffusion map theory [34, 35]. Denote the kernel function by

$$\begin{array}{c} K_{\varepsilon }\left(x,y\right)=\frac{1}{{\left(4\pi \varepsilon \right)}^{m/2}}e^{-\frac{\|x-y{\|}^{2}}{4\varepsilon }} \end{array},$$ (12)

where ***x*** and ***y*** are two samples in *m*-dimensional space, and *ε* is a parameter adjusting the kernel width. If there are *N* samples/cells {***x***_1_, ***x***_2_, …, ***x***_*N*_} in total obtained from probability density *r*(***x***), we can define

$$\begin{array}{cc} & q_{\varepsilon }\left(x_{i}\right)=\sum_{j=1}^{N}K_{\varepsilon }\left(x_{i},x_{j}\right), \end{array}$$ (13)

$$K_{\varepsilon ,\alpha }(x,y)=\frac{K_{\varepsilon }(x,y)}{q_{\varepsilon }^{\alpha }(x)q_{\varepsilon }^{\alpha }(y)},$$ (14)

$$\begin{array}{cc} & d_{\varepsilon ,\alpha }\left(x_{i}\right)=\sum_{j=1}^{N}K_{\varepsilon ,\alpha }\left(x_{i},x_{j}\right), \end{array}$$ (15)

and the transition matrix between samples *i* and *j* as

$$\begin{array}{c} P_{\varepsilon ,\alpha }\left(x_{i},x_{j}\right)=\frac{K_{\varepsilon ,\alpha }\left(x_{i},x_{j}\right)}{d_{\varepsilon ,\alpha }\left(x_{i}\right)}. \end{array}$$ (16)

The discrete backward Kolmogorov operator is constructed as

$$L_{\varepsilon ,\alpha }=\frac{P_{\varepsilon ,\alpha }-I}{\varepsilon }.$$ (17)

When the sample size *N* → ∞ and the kernel width *ε* goes to 0, the discrete operator *L*_*ε*,*α*_ tends to be an operator $L_{\alpha }$ in the continuous space. When *α* = 1/2, $L_{1/2}=\nabla \log r\left(x\right)\cdot \nabla +\Delta$ is exactly the backward operator in Eq (11) (see S1 Text for details). In our LDD algorithm, we first construct a weighted undirected k-nearest-neighbor (kNN) network by the similarity measure Eq (14) with the samples as nodes. After symmetrization, we obtain a Markov chain with transition matrix *P* = (*p*_*ij*_)_*N*×*N*_ through Eq (16) as

$$\begin{array}{c} p_{ij}=\begin{array}{c} P_{\varepsilon }{{}_{,}}_{\frac{1}{2}} \end{array}\left(x_{i},x_{j}\right),\;i,j=1,2,\ldots ,N. \end{array}$$ (18)

The stationary distribution of the Markov chain is defined as ***μ*** = (*μ*_*i*_)_1×*N*_, where

$$\begin{array}{c} \mu_{i}=\frac{d_{\varepsilon ,1/2}\left(x_{i}\right)}{\sum_{i=1}^{N}d_{\varepsilon ,1/2}\left(x_{i}\right)},\;i=1,2,\ldots ,N, \end{array}$$ (19)

which satisfies ***μ*** = ***μ*** *P*. *P* and ***μ*** are used in the procedures below. Usually for the Gaussian kernel, the bandwidth $\sqrt{2\varepsilon }$ is set as the median value of the distances between all samples.

### Clustering cells and computing the coarse-grained operator between clusters

There are lots of well-known clustering methods, such as k-means, spectral cluster, dynamical reduction [36], and new packages designed for single cell datasets, such as SC3 [44] and Seurat [45]. It is still a challenge to make a choice among the diverse approaches [46]. However, for our model, we have several limits when choosing a suitable clustering method. One important point is that different cell types should be separated into different groups. Each cell type holds a metastable state in the dynamical system. The other important point is that we need to maintain continuity of differentiation paths. Some nonlinear clustering approaches may fail, as they may scatter different clusters far away or eliminate the transition point that connects different branches. A suitable clustering is one fully using information from the data and coincident with the biological background. In our simulated datasets and real datasets, as the model is built on the original euclidean space, we used the linear principal component analysis (PCA) to reduce dimension and k-means to cluster the data, which has orthogonal invariance and keeps both the separation and continuity perfectly. When choosing the number of clusters in k-means, the following constrains should be considered. (1) From the expression of Eq (S7) in the S1 Text, we can conclude that the best number of the clusters is exactly the number of cell subtypes (the metastable states). The metastable wells are separated by the ridges of system potential *F*(***x***). Hence, there is an upper limit for the cluster number, which is decided by the number of metastable wells. (2) For the differentiation process with one lineage, the lower limit of cluster number is two, and for two lineages, it is four; one is for stem cells, one is around the bifurcation point, and the other two clusters stand for two branches. (3) If two neighboring clusters, which do not include the cluster near the bifurcation point, merge into one, Eq (S7) will still hold, and the pseudo-time order of the cells will not change. Under these constrains, we can ensure the LDD robustness for different cluster numbers. The results are shown in Fig J in S1 Text.

If we get *K* clusters of all cells by one of the above methods, we can define a coarse-grained matrix $\hat{P}\in R^{K\times K}$ between clusters *s* and *t* as

$$\begin{array}{c} {\hat{P}}_{st}=\hat{P}\left(\Omega_{s},\Omega_{t}\right)=\frac{{\hat{\mu }}_{t}}{n_{s}n_{t}}\sum_{i\in \Omega_{s}}\sum_{j\in \Omega_{t}}p_{ij},\;s,t=1,2,\ldots ,K, \end{array}$$ (20)

where ${\hat{\mu }}_{t}=\sum_{i\in \Omega_{t}}\mu_{i}$ and *n*_*s*_ is the number of samples in Ω_*s*_ (see S1 Text for details). Correspondingly, we can get the approximated operator $\hat{L}$ through $\hat{P}$ by Eq (17) as

$$\begin{array}{c} \hat{L}=\frac{\hat{P}-I}{\varepsilon }. \end{array}$$ (21)

### Theoretical derivation of net-flow for each cluster by divergence theorem

One of the most important advantages in the model is that we can compute the net-flow rate $\hat{R}$ of each cluster only based on scRNA-seq data. From Eq (1), if we only focus on the samples/cells in one cluster (or cell type) Ω_*s*_, we can define the conditional probability density function (cpdf) as

$$r_{s}\left(x,t\right)=\frac{c\left(x,t\right)\chi_{\Omega_{s}}(x)}{\int_{\Omega_{s}}c\left(x,t\right)\text{d}x},$$ (22)

where $\chi_{\Omega_{s}}\left(x\right)$ is the indicator function of Ω_*s*_, i.e. $\chi_{\Omega_{s}}\left(x\right)=1$ when ***x*** is in Ω_*s*_ and 0 otherwise. In the long time limit, *c*(***x***, *t*) and *r*_*s*_(***x***, *t*) will converge to the steady distribution *p*(***x***) and *r*_*s*_(***x***), respectively. By applying the divergence theorem to the equation satisfied by *r*_*s*_(***x***) and eliminating equal terms, we can derive an equation satisfied by the net-flow rate ${\hat{R}}_{s}$ of cluster *s* as

$$\begin{array}{c} {\hat{R}}_{s}≜\int_{\Omega_{s}}R\left(x\right)r_{s}\left(x\right)\;dx=-D\int_{\Omega_{s}}\Delta r_{s}\left(x\right)\;dx. \end{array}$$ (23)

The derivation details are shown in S1 Text.

### Numerical computation of net-flow for each cluster

The net-flow rate formula can be simplified by marginal density functions as

$$\begin{array}{cc} {\hat{R}}_{s} & =-D\int_{\Omega_{s}}\Delta r_{s}\left(x\right)\;dx=-D\sum_{j=1}^{m}\int_{\Omega_{s}^{\left(j\right)}=\left[a_{s}^{\left(j\right)},b_{s}^{\left(j\right)}\right]}\partial_{x}^{2}r_{s}^{\left(j\right)}\left(x\right)\;dx\\ & =-D\sum_{j=1}^{m}[\partial_{x}r_{s}^{\left(j\right)}\left(b_{s}^{\left(j\right)}\right)-\partial_{x}r_{s}^{\left(j\right)}\left(a_{s}^{\left(j\right)}\right)],\;s=1,2,\ldots ,K, \end{array}$$ (24)

where ***x*** = (*x*^(1)^, *x*^(2)^, …, *x*^(*m*)^)^*T*^ ∈ Ω_*s*_, $r_{s}^{\left(j\right)}\left(x\right)$ is the marginal density of *r*_*s*_(***x***) on the *x*^(*j*)^-axis, and $\Omega_{s}^{\left(j\right)}=\left[a_{s}^{\left(j\right)},b_{s}^{\left(j\right)}\right]$ is the interval that $r_{s}^{\left(j\right)}\left(x\right)$ lies in. By approximating $r_{s}^{\left(j\right)}\left(x\right)$ in one-dimensional space through the kernel method and summation of the boundary derivatives, we can compute ${\hat{R}}_{s}$ conveniently.

When computing ${\hat{R}}_{s}$ for each cluster separately by Eq (24), the steady state Eq (5) of the original system may break down due to finite sample size effect and the numerical error in discrete computation. A post-processing of ${\hat{R}}_{s}$ is needed to ensure that the system is steady in discrete setting. By integrating Eq (1) in the whole space, letting *t* → ∞ and using Eqs (22) and (23), we obtain the equation for ${\hat{R}}_{s}$ as follows:

$$\begin{array}{c} \sum_{s=1}^{K}\left({\hat{R}}_{s}\cdot \int_{\Omega_{s}}c\left(x\right)\;dx\right)=0. \end{array}$$ (25)

If there are *n*_*s*_ samples in cluster *s* and $N=\sum_{s=1}^{K}n_{s}$, the constraint Eq (25) can be written as

$$\begin{array}{c} \sum_{s=1}^{K}n_{s}{\hat{R}}_{s}=0. \end{array}$$ (26)

Then we post-process the results obtained from Eq (24) as

$${\tilde{R}}_{s}={\hat{R}}_{s}-\frac{\sum_{s=1}^{K}n_{s}{\hat{R}}_{s}}{N},$$ (27)

in order to satisfy Eq (26). In other parts of this paper, for notational convenience, we still use ${\hat{R}}_{s}$ instead of ${\tilde{R}}_{s}$, but note that it is a post-processed value.

### Computing potential and constructing differentiation paths of cells

By Eqs (10) and (21), the potential $\hat{V}$ in the cluster level satisfies $\hat{L}\hat{V}=-\hat{R}$, i.e.

$$\begin{array}{c} \left(\hat{P}-I\right)\hat{V}=-\hat{R}\varepsilon , \end{array}$$ (28)

where $\hat{P}$ is the coarse-grained matrix between clusters, vector $\hat{V}={\left({\hat{V}}_{1},{\hat{V}}_{2},\ldots ,{\hat{V}}_{K}\right)}^{T}$ is the LDD potential of different clusters, and vector $\hat{R}={\left({\hat{R}}_{1},{\hat{R}}_{2},\ldots ,{\hat{R}}_{K}\right)}^{T}$ represents the net-flow of each cluster. As the matrix on the left hand side of Eq (28) is degenerate, we need to compute its pseudo-inverse and get the least square solution of $\hat{V}$ as

$$\begin{array}{c} \hat{V}=-{\left(\hat{P}-I\right)}^{†}\hat{R}\varepsilon . \end{array}$$ (29)

On the other hand, the structure of differentiation branches can be inferred from the weight matrix $\tilde{P}\in R^{K\times K}$ between clusters, whose element is given by

$$\begin{array}{c} {\tilde{p}}_{st}=\sum_{i\in \Omega_{s}}\sum_{j\in \Omega_{t}}\mu_{i}p_{ij},\;s,t=1,2,\ldots ,K, \end{array}$$ (30)

and $\tilde{P}$ will become the transition matrix between clusters after row normalization [36]. Nonzero elements in $\tilde{P}$ represent differentiation paths, while in some cases we use a threshold to eliminate small values. The direction of differentiation is determined by $\hat{V}$ from high to low potential. Thus, using $\tilde{P}$ and $\hat{V}$, we can construct the whole differentiation landscape or picture only by the gene expression matrix obtained from an scRNA-seq dataset. Note that the noise *D* is not required for evaluating the potential landscape *V* of cells.

### Drawing the illustrative landscape

For most of the datasets in this paper, we constructed an illustrative 3D landscape. It is plotted by fitting functions

$$\begin{array}{cc} f\left(x,y\right) & =\frac{1}{N}\sum_{i=1}^{N}\left(-e^{-\frac{{\left(x-x_{i}\right)}^{2}+{\left(y-y_{i}\right)}^{2}}{{\left(\sigma /{\tilde{p}}_{i}\right)}^{2}}}\right),\\ V\left(x,y\right) & =af\left(x,y\right)+bg\left(x,y\right), \end{array}$$ (31)

where (*x*_*i*_, *y*_*i*_) are the position of *N* samples in the two-dimensional reduced space, ${\tilde{p}}_{i}$ equals to ${\tilde{P}}_{ss}$ if sample *i* belongs to cluster *s*, *g*(*x*, *y*) is a function (usually linear) positively correlated with $\hat{V}$, and *a*, *b*, *σ* are adjustable parameters. Some high values could be set as *NaN* when plotting.

## Supporting information

S1 Text Supplementary information of this paper. The supplementary document provides one algorithm, six notes, and ten supplementary figures for the main text.
(PDF)
