# Supplementary material for: Quantifying pluripotency landscape of cell differentiation from scRNA-seq data by continuous birth-death process
Source: PLoS Comput Biol. 2019 Nov 13;15(11):e1007488. doi: 10.1371/journal.pcbi.1007488 (PMC6876891; doi:10.1371/journal.pcbi.1007488)
Supplement: S1 Text — The supplementary document provides one algorithm, six notes, and ten supplementary figures for the main text. (PDF) [file pcbi.1007488.s001.pdf]

Supplementary Information for "Quantifying pluripotency landscape of cell differentiation from scRNA-seq data by continuous birth-death process"

Jifan Shi,[1] Tiejun Li,[2,*] Luonan Chen,[3,4,5,†] and Kazuyuki Aihara[1,6,‡]

[1]*Institute of Industrial Science, The University of Tokyo, 153-8505 Tokyo, Japan*
[2]*LMAM and School of Mathematical Sciences, Peking University, Beijing 100871, China*
[3]*Key Laboratory of Systems Biology, Center for Excellence in Molecular Cell Science, Institute of Biochemistry and Cell Biology, Chinese Academy of Sciences, 200031 Shanghai, China*
[4]*Center for Excellence in Animal Evolution and Genetics, Chinese Academy of Sciences, 650223 Kunming, China*
[5]*School of Life Science and Technology, ShanghaiTech University, 201210 Shanghai, China*
[6]*International Research Center for Neurointelligence, The University of Tokyo Institutes for Advanced Study, The University of Tokyo, 113-0033 Tokyo, Japan*

This supplementary document includes:

Section S1: Supplementary Algorithm
Section S2: Supplementary Notes

- Note S1: Details of simulation models
- Note S2: Description of real datasets
- Note S3: Details on the approximation of the backward operator $\mathcal{L}$
- Note S4: Details on the approximation of the net-flow R
- Note S5: Details on the derivation of the coarse-grained matrix $\hat{P}$
- Note S6: Description of other pseudo-time methods

Section S3: Supplementary Figures

- Figure A: Supplementary figures of simulation datasets.
- Figure B: Supplementary figures of real datasets.
- Figure C: TSCAN results of seven datasets.
- Figure D: Monocle2 results of seven datasets.
- Figure E: DiffusionMap results of seven datasets.
- Figure F: DPT results of seven datasets.
- Figure G: SLICER results of seven datasets.
- Figure H: Slingshot results of seven datasets.
- Figure I: Detailed flowchart of the LDD procedure.
- Figure J: LDD results under different cluster numbers.

*Electronic address: tieli@pku.edu.cn
†Electronic address: lnchen@sibs.ac.cn
‡Electronic address: aihara@sat.t.u-tokyo.ac.jp

S1. SUPPLEMENTARY ALGORITHM

Algorithm 1 Framework of LDD to estimate the cell differentiation landscape from scRNA-seq data.

Input:

A preprocessed dataset matrix $X \in \mathbb{R}^{n\times m}$, where n is the number of samples and m is the number of genes;

Output:

The number of clusters, K;

The cell type (cluster indices) for each sample, C;

The weight matrix between clusters, $\tilde{P}$;

The potential value for each cell type, $\hat{V}$;

Steps:

1: Construct a k-nearest-neighbor (kNN) graph with the data matrix X;

2: On the kNN graph, compute the diffusion distance matrix between samples, and construct a Markov chain between nodes with a transition matrix named P;

3: Apply a suitable clustering method to cluster samples into K groups, and note the cluster indices of each sample as C;

4: Generate the coarse-grained matrix $\hat{P}$, which represents the transfer between clusters, and the weight matrix $\tilde{P}$, which denotes the transition path between clusters;

5: Compute the net-flow $\hat{R}$ for each cluster;

6: Compute the pseudo-time potential $\hat{V}$ of each cluster by $\hat{P}$ and $\hat{R}$;

7: **return** $K, C, \hat{V}, \tilde{P}$.

Notes:

If we have suitable clustering of samples in advance, the number of clusters K and the cell type C can be set as inputs, and Step 3 can be omitted. A detailed flowchart can be found in Fig. I.

The codes of LDD and the pre-processed datasets used in the paper can be downloaded from https://github.com/smsxiaomayi/LDD/blob/master/LDDcode.zip.

S2. SUPPLEMENTARY NOTES

Note S1. Details of simulated models

The drift-diffusion process evolves as

$$\boldsymbol{x}(t+\Delta t) = \boldsymbol{x}(t) - \nabla F(\boldsymbol{x}(t))\cdot \Delta t + \sqrt{D\Delta t}\cdot \boldsymbol{\xi}(t),$$

where $\boldsymbol{x} = (x_1, x_2, \ldots, x_{50})^T$ is the position of a particle in 50 dimensions, F is a potential function, D is the noise amplitude, and $\boldsymbol{\xi}$ is a normal random vector standing for noise. We choose

$$F(\boldsymbol{x}) = \frac{x_1^4}{4} + \frac{x_1^2 x_2}{2} + \frac{x_2}{6},$$

$\Delta t = 0.001, D = 0.04, \xi_i \sim N(0,1), i = 1,2,$ and $\xi_j \sim N(0, 1/4), j \geq 3$. Domain $\{\boldsymbol{x} | -0.05 < x_1 < 0.05, x_2 = 1\}$ is set as the source region, where particles are added uniformly, and the exit region is set as $\{\boldsymbol{x}|x_2 \leq -2\}$, where particles disappear once crossing $x_2 = -2$. A reflection boundary is set at $\{\boldsymbol{x}|x_2 = 1\}$. If n particles exit at time t, n new particles are added in the source region to maintain a steady system. In total, 400 particles were simulated from $t = 0$ to $t = 10^5$. In preprocessing, principal component analysis (PCA) was used to reduce the dimension into two. Subsequently, we scaled the data to make the maximum absolute value of its elements to 1 (So are the following other examples). For this dataset, the second principal component was taken as the true differentiation time.

For the two-gene regulatory network shown in Fig. 2C, where two genes inhibit each other, we simulated the data with the following rules:

1. x_i decreases with the rate $0.003x_i, i = 1, 2$.

2. x_i increases with the rate $\max\{\frac{(0.01x_i)^4}{1+(0.01x_i)^4} - \frac{(0.003x_j)^4}{1+(0.003x_j)^4}, 0\}, \quad i, j \in \{1, 2\}, i \neq j$.

3. The source point is $(x_1, x_2) = (225, 225)$, and an absorbed boundary condition is set at $\{\boldsymbol{x}|x_i = 0, i = 1, 2\}$, which represent cell birth and death, respectively. If n cells die at time t, the same number of cells will be born from the source.

4. τ-leaping algorithm is used for the simulation with the step size $\tau = 0.5$ and final time $T = 5 \times 10^4$. $N = 1000$ samples are collected.

For this two-gene network, $-\min\{x_1, x_2\}$ is set as the measure of the true time direction.

For the six-gene regulatory network shown in Fig. 2D, six genes evolve using the following rules:

1. x_i decreases with the rate $0.005, i = 1, 2, \ldots, 6$.
2. x_i increases with the rate $\frac{5}{1+(x_j/200)^2}$, where $i, j \in \{1, 2\}, i \neq j$.
3. x_i increases with the rate $\frac{x_1^{20}}{x_1^{20}+700^{20}} \cdot \frac{5}{1+(x_j/200)^2}$, where $i, j \in \{3, 4\}, i \neq j$.
4. x_i increases with the rate $\frac{x_2^{20}}{x_2^{20}+700^{20}} \cdot \frac{5}{1+(x_j/200)^2}$, where $i, j \in \{5, 6\}, i \neq j$.
5. The system is simulated by the stochastic differentiation equation with white noise. Noise amplitude is 1.3, and the time interval is from $t = 0$ to $T = 5 \times 10^4$ with the step size $\Delta t = 1$.
6. 1000 cells are simulated. Gene expression in the new cell is sampled from a normal distribution with mean $\boldsymbol{x} = (500, 500, 40, 40, 40, 40)$ and standard derivation 10 (samples with negative element will be resampled). If n samples cross $\{\boldsymbol{x}|x_i = 0, i = 1, 2, \ldots, 6\}$, they will be removed, and n new samples are added from the source.

There are four branches. An increasing in four cases — (x_1, x_3) or (x_1, x_4) or (x_2, x_5) or (x_2, x_6), will occur, and in each case, the other four genes are few. For the six-gene example in this paper, we use $-\min\{x_1, x_2\}$ as the measure of the true time direction.

Note S2. Description of real datasets

Guo's dataset was derived from [3]. In total, 46 selected genes and 438 samples from mouse stem cells, consisting of 7 stages named oocyte, 2-cell, 4-cell, 8-cell, morula, E3.5 blastocyst, and E4.25 blastocyst were included. Further, three types, including trophectoderm (TE), primitive endoderm (PE), and epiblast (EPI) were formed during the differentiation process. The PE and EPI made up the inner cell mass (ICM) and were almost distinguishable. In our example, we observed two branches: TE and ICM. We conducted PCA to reduce to three dimensions, and four groups clustered by k-means. Figures B(a-b) show two groups on the main branch and other two groups belonging to two sublineages.

Nef's dataset was reported in [8], which used XY mouse gonads to study mouse sex determination. It is accessible from GEO: *GSE97519*. In total, 400 cells passed the quality control testing in [8]. After log-normalization, 8210 important genes were chosen, which had standard derivations larger than 1. It consisted of five stages named E10.5, E11.5, E12.5, E13.5, and E16.5. The interstitial progenitor cells and Sertoli cells formed two branches; however, we removed the fetal Leydig cell lineage, which consisted of very few samples. In our example, after reducing to two dimensions by PCA, we clustered the samples into five groups and computed their potential values (Figs. B(c-e)).

Xu's dataset originated from [11], which is accessible from GEO: *GSE90047*. The original data contained 447 samples and 40824 genes from the mouse embryo. After log-normalization and gene selection, 1140 effective genes, which showed expression in all samples, were detected. Seven stages from E10.5 to E17.5 described bipotential hepatoblasts differentiation into hepatocytes and cholangiocytes. Five clusters were found. The potential values in two-dimensional PCA space and differentiation paths are shown in Figs. 3A-C.

Furlan's dataset was derived from [2], which is accessible by GEO: *GSE99933*. In brief, 337 cells revealed that Schwann cell precursors (SCPs), a type of peripheral glial stem cell, had the ability to differentiate into large numbers of chromaffin cells in the adrenal medulla. Further, 500 genes, which highly correlated with *Chga*, a characteristic gene in the differentiation process, were selected. In our example, 4 clusters formed in the two-dimensional PCA

space. A linear process from SCPs to chromaffin cells through a moderate differentiation bridge could be detected, as shown in Figs. 3D-F.

A summary of information derived from real datasets is listed in Table A.

Table A: Information of the real datasets

	Guo	Nef	Xu	Furlan
Ref.	[3]	[8]	[11]	[2]
database	MGI-J:140465	GSE97519	GSE90047	GSE99933
scRNA-Seq	TaqMan	C1 Autoprep System	Smart-seq2	Smart-seq2
sample size	438	400	447	337
gene size	46	8210	1140	500
number of branches	2	2	2	1

Note S3. Details on the approximation of the backward operator $\mathcal{L}$

For the operator $L_{\varepsilon,\alpha}$ defined by eqn. (17), we will prove that under the large sample limit $N \to \infty$, it will converge to a continuous backward operator, i.e.

$$\mathcal{L}_\alpha = \lim_{\varepsilon \to 0} L_{\varepsilon,\alpha} = 2(1-\alpha)\nabla \log r \cdot \nabla + \Delta.$$

Proof: The large sample limit $N \to \infty$ is the basic assumption. Under this assumption and the Laplacian approximation

$$\int K_\varepsilon(\boldsymbol{x}, \boldsymbol{y})\phi(\boldsymbol{y})\,\mathrm{d}\boldsymbol{y} = \phi(\boldsymbol{x}) + \varepsilon\Delta\phi(\boldsymbol{x}) + o(\varepsilon),$$

where $\phi(\boldsymbol{x})$ is any smooth function, we can approximate eqns. (13) and (15) as

$$q_\varepsilon(\boldsymbol{x}) = \int K_\varepsilon(\boldsymbol{x}, \boldsymbol{y})r(\boldsymbol{y})\,\mathrm{d}\boldsymbol{y} = r(\boldsymbol{x}) + \varepsilon\Delta r(\boldsymbol{x}) + o(\varepsilon),$$

and

$$d_{\varepsilon,\alpha}(\boldsymbol{x}) = \int K_{\varepsilon,\alpha}(\boldsymbol{x}, \boldsymbol{y})r(\boldsymbol{y})\,\mathrm{d}\boldsymbol{y} = q_\varepsilon^{-\alpha}(\boldsymbol{x})r^{1-\alpha}(\boldsymbol{x})\left[1 - \varepsilon\alpha\frac{\Delta r(\boldsymbol{x})}{r(\boldsymbol{x})} + \varepsilon\frac{\Delta r^{1-\alpha}(\boldsymbol{x})}{r^{1-\alpha}(\boldsymbol{x})} + o(\varepsilon)\right].$$

The shift operator, which is the limitation of the transition matrix eqn. (16) for infinite samples, has the asymptotic expansion as

$$T_{\varepsilon,\alpha}\phi(\boldsymbol{x}) \triangleq \int P_{\varepsilon,\alpha}(\boldsymbol{x}, \boldsymbol{y})\phi(\boldsymbol{y})r(\boldsymbol{y})\,\mathrm{d}\boldsymbol{y} = \phi(\boldsymbol{x}) + \varepsilon\left[\frac{\Delta(r^{1-\alpha}(\boldsymbol{x})\phi(\boldsymbol{x}))}{r^{1-\alpha}(\boldsymbol{x})} - \phi(\boldsymbol{x})\frac{\Delta r^{1-\alpha}(\boldsymbol{x})}{r^{1-\alpha}(\boldsymbol{x})}\right] + o(\varepsilon).$$

Thus, we have the infinitesimal backward operator as

$$\mathcal{L}_\alpha\phi(\boldsymbol{x}) = \lim_{\varepsilon \to 0} L_{\varepsilon,\alpha}\phi(\boldsymbol{x}) = \lim_{\varepsilon \to 0}\frac{T_{\varepsilon,\alpha} - I}{\varepsilon}\phi(\boldsymbol{x}) = 2(1-\alpha)\nabla \log r(\boldsymbol{x}) \cdot \nabla\phi(\boldsymbol{x}) + \Delta\phi(\boldsymbol{x}).$$

■

If we define $\mathcal{L}_\alpha^*$ as the conjugate operator of $\mathcal{L}_\alpha$ under the weighted inner product $\langle\cdot,\cdot\rangle_r$, with the same procedure above we obtain

$$\mathcal{L}_\alpha^*\phi(\boldsymbol{x}) = \lim_{\varepsilon \to 0} L_{\varepsilon,\alpha}^T\phi(\boldsymbol{x}) = (2\alpha - 1)\frac{\Delta r(\boldsymbol{x})}{r(\boldsymbol{x})}\phi(\boldsymbol{x}) + 2\alpha\frac{\nabla r(\boldsymbol{x})}{r(\boldsymbol{x})} \cdot \nabla\phi(\boldsymbol{x}) + \Delta\phi(\boldsymbol{x}),$$

where $L_{\varepsilon,\alpha}^T$ is the transpose of $L_{\varepsilon,\alpha}$. $\mathcal{L}_\alpha^*$ is also called the infinitesimal forward operator. Some interesting cases include: when $\alpha = 1$, $\mathcal{L}_1 = \Delta$, which is the Laplace-Beltrami operator; when $\alpha = 1/2$, $\mathcal{L}_{1/2} = \mathcal{L}_{1/2}^* = \nabla \log r(x) \cdot \nabla + \Delta$, which is exactly the backward operator in eqn. (11).

Note S4. Details on the approximation of the net-flow R

In this note, the method to approximate the net-flow for each cluster is described.

First, we list the original continuous birth-death process, i.e.

$$\frac{\partial c(\boldsymbol{x},t)}{\partial t} = \nabla \cdot (c(\boldsymbol{x},t)\nabla F(\boldsymbol{x})) + D\Delta c(\boldsymbol{x},t) + R(\boldsymbol{x})c(\boldsymbol{x},t), \tag{S1}$$

where $\boldsymbol{x}$ is a vector representing the gene expression, $c(\boldsymbol{x},t)$ is the probability density function (pdf) of cells at $\boldsymbol{x}$, $F(\boldsymbol{x})$ is a potential function, D is the noise amplitude, and $R(\boldsymbol{x})$ is the net-flow of cells at state $\boldsymbol{x}$. $\nabla, \nabla\cdot$ and Δ denote the gradient, divergence, and Laplace operators, respectively. With the conditional probability density function (cpdf)

$$r_s(\boldsymbol{x},t) = \frac{c(\boldsymbol{x},t)\chi_{\Omega_s}(\boldsymbol{x})}{\int_{\Omega_s} c(\boldsymbol{x},t)\,\mathrm{d}\boldsymbol{x}}, \tag{S2}$$

we obtain the evolution of $r_s(\boldsymbol{x},t)$ as

$$\begin{aligned}\frac{\partial r_s(\boldsymbol{x},t)}{\partial t} =& \mathcal{L}^* r_s(\boldsymbol{x},t) + R(\boldsymbol{x})r_s(\boldsymbol{x},t) \\ & - \left(\int_{\Omega_s} [\nabla \cdot (r_s(\boldsymbol{x},t)\nabla F(\boldsymbol{x})) + D\Delta r_s(\boldsymbol{x},t) + R(\boldsymbol{x})r_s(\boldsymbol{x},t)]\,\mathrm{d}\boldsymbol{x}\right) \cdot r_s(\boldsymbol{x},t),\end{aligned} \tag{S3}$$

where

$$\mathcal{L}^* f(\boldsymbol{x}) = \nabla \cdot (\nabla F(\boldsymbol{x}) f(\boldsymbol{x})) + \Delta f(\boldsymbol{x}) \tag{S4}$$

is the forward operator.

In the long time limit, $c(\boldsymbol{x},t)$ and $r_s(\boldsymbol{x},t)$ will converge to the steady distribution $p(\boldsymbol{x})$ and $r_s(\boldsymbol{x})$, respectively. When $t \to \infty$, using

$$r_s(\boldsymbol{x}) = \frac{p(\boldsymbol{x})\chi_{\Omega_s}(\boldsymbol{x})}{\int_{\Omega_s} p(\boldsymbol{x})\,\mathrm{d}\boldsymbol{x}} \tag{S5}$$

and eqn. (S1), we get

$$\frac{\partial r_s(\boldsymbol{x})}{\partial t} = 0, \quad \text{and} \quad \mathcal{L}^* r_s(\boldsymbol{x}) + R(\boldsymbol{x})r_s(\boldsymbol{x}) = 0. \tag{S6}$$

Assuming that samples have separated into different metastable states, $\nabla F(\boldsymbol{x})$ and $\mathrm{d}\boldsymbol{w}$ are orthogonal, where $\mathrm{d}\boldsymbol{w}$ is the outer normal vector on the boundary of Ω_s. We can also obtain

$$\int_{\Omega_s} \nabla \cdot (r_s(\boldsymbol{x})\nabla F(\boldsymbol{x}))\,\mathrm{d}\boldsymbol{x} = \int_{\partial\Omega_s} r_s(\boldsymbol{w})\nabla F(\boldsymbol{w}) \cdot \mathrm{d}\boldsymbol{w} = 0 \tag{S7}$$

by the divergence theorem.

Finally, after we combine eqns. (S6) and (S7), and let $t \to \infty$ in eqn. (S3), the net-flow rate $\hat{R}_s$ of cluster s can be calculated as

$$\hat{R}_s \triangleq \int_{\Omega_s} R(\boldsymbol{x})r_s(\boldsymbol{x})\,\mathrm{d}\boldsymbol{x} = -D\int_{\Omega_s} \Delta r_s(\boldsymbol{x})\,\mathrm{d}\boldsymbol{x}. \tag{S8}$$

In the aspect of numerical computation, we know that samples $\{\boldsymbol{x}_1, \boldsymbol{x}_2, \ldots, \boldsymbol{x}_N\} \in \Omega_s$ have cpdf $r_s(\boldsymbol{x})$. When the sample size is $N \to \infty$, the limitations

$$\begin{aligned}\min\{x_1^{(j)}, x_2^{(j)}, \ldots, x_N^{(j)}\} &\to a_s^{(j)}, \\ \max\{x_1^{(j)}, x_2^{(j)}, \ldots, x_N^{(j)}\} &\to b_s^{(j)},\end{aligned}$$

hold, where $\boldsymbol{x}_i = (x_i^{(1)}, x_i^{(2)}, \ldots, x_i^{(m)})^T$, $\Omega_s^{(j)} = [a_s^{(j)}, b_s^{(j)}]$ is the support set of $r_s^{(j)}(x)$, and $r_s^{(j)}(x)$ is the marginal density of $r_s(\boldsymbol{x})$ on the $x^{(j)}$-axis. Thus, the net-flow rate formula eqn. (S8) can be simplified by marginal density functions as

$$\begin{aligned}\hat{R}_s &= -D\int_{\Omega_s} \Delta r_s(\boldsymbol{x})\,\mathrm{d}\boldsymbol{x} = -D\sum_{j=1}^{m}\int_{\Omega_s^{(j)}=[a_s^{(j)},b_s^{(j)}]} \partial_x^2 r_s^{(j)}(x)\,\mathrm{d}x \\ &= -D\sum_{j=1}^{m}\left[\partial_x r_s^{(j)}(b_s^{(j)}) - \partial_x r_s^{(j)}(a_s^{(j)})\right], \qquad s = 1, 2, \ldots, K. \end{aligned} \tag{S9}$$

Thus, by approximating $r_s^{(j)}(\boldsymbol{x})$ in the one-dimensional space through the kernel method, and adding up all the boundary derivatives, we can compute $\hat{R}_s$ conveniently.

Note S5. Details on the derivation of the coarse-grained matrix $\hat{\mathbf{P}}$

To approximate a coarse-grained transfer operator $\hat{P} = (\hat{p}_{st})_{K\times K}$ on the clusters space, we drive from the ϵ-approximation equation

$$\mathcal{L}_{\varepsilon,\frac{1}{2}} V = \frac{T_{\varepsilon,\frac{1}{2}} - I}{\varepsilon} V(\boldsymbol{x}) = -R(\boldsymbol{x}), \tag{S10}$$

where $T_{\varepsilon,\frac{1}{2}}$ is the transfer operator satisfying

$$T_{\varepsilon,\frac{1}{2}}\phi(\boldsymbol{x}) = \int_{\boldsymbol{y}\in\mathbb{R}^m} P_{\varepsilon,\frac{1}{2}}(\boldsymbol{x},\boldsymbol{y})\phi(\boldsymbol{y})p(\boldsymbol{y})\,\mathrm{d}\boldsymbol{y},$$

and $p(\boldsymbol{x})$ is the steady distribution. Integrating both sides of eqn. (S10) with $r_s(\boldsymbol{x})$ over Ω_s, and using the definition of $\hat{R}_s$ in eqn. (S8), we obtain

$$\int_{\boldsymbol{x}\in\Omega_s}\int_{\boldsymbol{y}\in\mathbb{R}^m} P_{\varepsilon,\frac{1}{2}}(\boldsymbol{x},\boldsymbol{y})V(\boldsymbol{y})p(\boldsymbol{y})r_s(\boldsymbol{x})\,\mathrm{d}\boldsymbol{x}\,\mathrm{d}\boldsymbol{y} - \int_{\boldsymbol{x}\in\Omega_s} V(\boldsymbol{x})r_s(\boldsymbol{x})\,\mathrm{d}\boldsymbol{x} = -\varepsilon\hat{R}_s. \tag{S11}$$

By defining the piecewise constant potential $\hat{V}_s$ and distribution $\hat{\mu}_s$ over the cluster space as

$$\hat{V}_s \triangleq \int_{\boldsymbol{x}\in\Omega_s} V(\boldsymbol{x})r_s(\boldsymbol{x})\,\mathrm{d}\boldsymbol{x}, \quad \hat{\mu}_s \triangleq \int_{\boldsymbol{x}\in\Omega_s} p(\boldsymbol{x})\,\mathrm{d}\boldsymbol{x},$$

the eqn. (S11) can be approximated by

$$\left(\sum_t \int_{\boldsymbol{x}\in\Omega_s}\int_{\boldsymbol{y}\in\Omega_t} P_{\varepsilon,\frac{1}{2}}(\boldsymbol{x},\boldsymbol{y})r_s(\boldsymbol{x})r_t(\boldsymbol{y})\,\mathrm{d}\boldsymbol{x}\,\mathrm{d}\boldsymbol{y}\cdot\hat{\mu}_t\cdot\hat{V}_t\right) - \hat{V}_s = -\varepsilon\hat{R}_s. \tag{S12}$$

After we apply the diffusion map theory on N samples with transition matrix $p_{ij} = P_{\varepsilon,1/2}(\boldsymbol{x}_i, \boldsymbol{x}_j)$ and compute

$$\hat{p}_{st} \triangleq \frac{\hat{\mu}_t}{n_s n_t}\sum_{i\in\Omega_s}\sum_{j\in\Omega_t} p_{ij}, \tag{S13}$$

where n_s is the number of samples in Ω_s, we can derive the discrete numerical equation of eqn. (S12) as

$$\sum_{t=1}^{K}\hat{p}_{st}\hat{V}_t - \hat{V}_s = -\varepsilon\hat{R}_s, \quad s = 1, 2, \ldots, K,$$

or its matrix version

$$\frac{\hat{P} - I}{\epsilon}\hat{V} = -\hat{R}, \tag{S14}$$

where $\hat{V} = (\hat{V}_1, \hat{V}_2, \cdots, \hat{V}_K)^T$ and $\hat{R} = (\hat{R}_1, \hat{R}_2, \cdots, \hat{R}_K)^T$. Eqns. (S13) and (S14) are required for eqns. (20) and (28) in the main text.

Note S6. Description of other pseudo-time methods

Six popular pseudo-time algorithms are compared in this study. Below, we provide a short description and list some notes on their applications. Corresponding figures are included in the following supplemental document.

TSCAN [6] ("Tools for Single Cell Analysis") first clusters cells into groups and then constructs a minimum spanning tree (MST). The pseudo-time of TSCAN is determined by the order of samples' projections on the MST. The number of clusters is needed as the input. When applying TSCAN to our datasets, we used the same number of clusters as LDD, except using five clusters for Simu2. The pseudo-times of some samples were not derived by TSCAN. Therefore, we only considered those with pseudo-times for comparison. The two-dimensional plots for every dataset are shown in Fig. C. TSCAN has PCA plot different from LDD or Slingshot due to additional scaling applied on the dataset.

Monocle2 [7] uses the reversed graph embedding (RGE) technique to learn a graph's structure. A root cell is needed as one of the inputs, and the pseudo-time is calculated by the geodesic distance between the projections on the tree structure. Therefore, choosing the distribution family for the gene expression matrix is important. As Monocle2 does log-normalization in the algorithm by itself, we used "exp(data)" as the input and chose the distribution family "tobit()", except for Simu2 and Simu3 which used "data" as the input along with the parameter "negbinomial.size()". The results are shown in Fig. D.

Diffusion map [1, 4] and DPT [5] are two similar methods. The former uses the first coordinate of the diffusion map's reduction as the pseudo-time, and the latter computes the so-called "Diffusion pseudotime" by the accumulation of the transition matrix. Duplicated samples must be eliminated, and a root cell is needed for DPT. Figures E–F show the corresponding results for the two algorithms on seven datasets.

SLICER [10] uses the local linear embedding (LLE) for dimension reduction. A kNN graph is constructed, and the shortest path distance from a root cell on the graph is considered as the pseudo-time. The final results of SLICER for seven datasets are shown in Fig. G.

Slingshot [9] is a recently published method. Slingshot can fit a principal curve for each lineage obtained by MST to generate the pseudo-time. Figure H shows the results of Slingshot for the seven datasets.

Among the six algorithms, if a root cell as the start node is needed, the first one with the smallest true time label in the samples will be chosen. Some of the six algorithms may generate pseudo-times that are negatively correlated with the true time labels, because they could not automatically determine the correct direction. In that case, a minus sign is added to the pseudo-times to create a positive correlation.

S3. SUPPLEMENTARY FIGURES

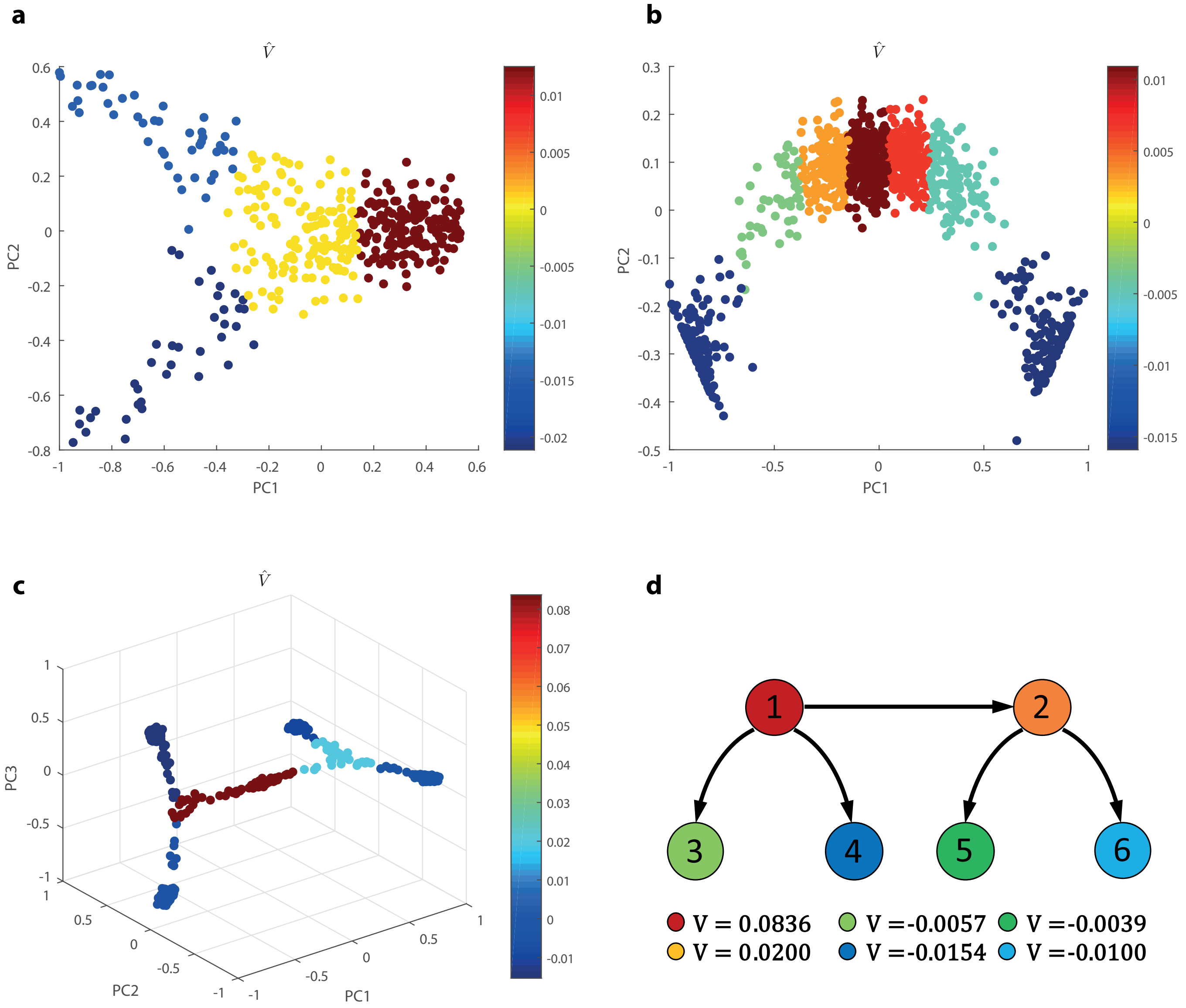

Figure A: **Supplementary figures of simulation datasets.** **(a)** is the 2D potential plot of the samples from the drift-diffusion model. **(b)** is the 2D potential plot of the samples from the two-gene regulatory network. **(c)** is the 3D potential plot of the samples from the six-gene regulatory network. In **(a-c)**, the color represents the value of $\hat{V}$ computed by LDD, in which 4, 7, and 6 cell types are clustered, respectively. **(d)** is the differentiation paths between the six clusters in the six-gene network.

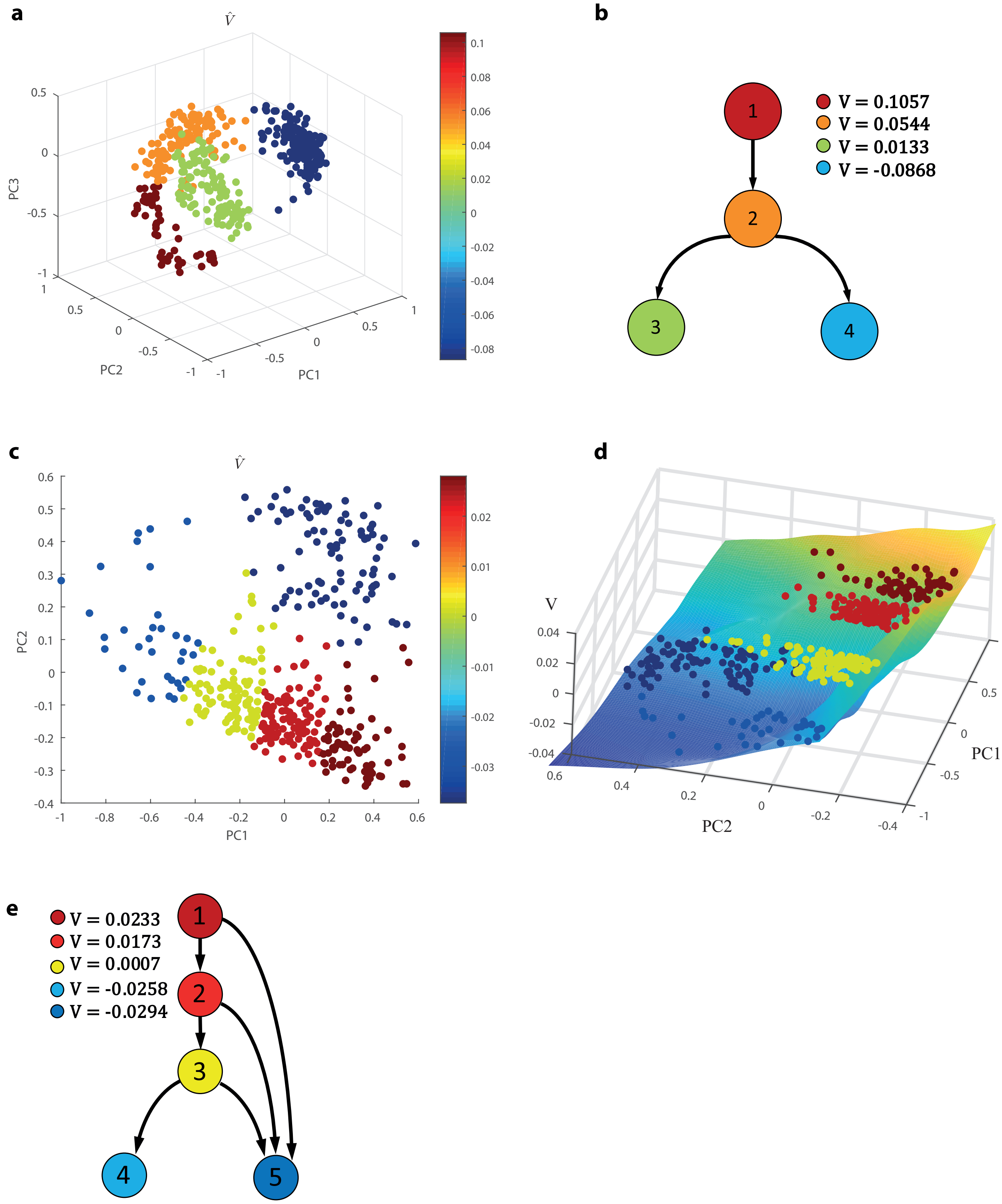

Figure B: **Supplementary figures of real datasets.** **(a)** shows the potential values of Guo's dataset in the three-dimensional PCA space. **(b)** shows the differentiation paths of Guo's dataset, which describe embryo growth from oocytes to TE or ICM. **(c-e)** are the results of Nef's dataset by LDD. **(c)** shows the potential value in the two-dimensional reduced PCA space. **(d)** adds an illustrative landscape based on **(c)**, and **(e)** shows the differentiation paths. In **(e)**, cluster 4 is the interstitial progenitor cell lineage and cluster 5 is the Sertoli cell lineage.

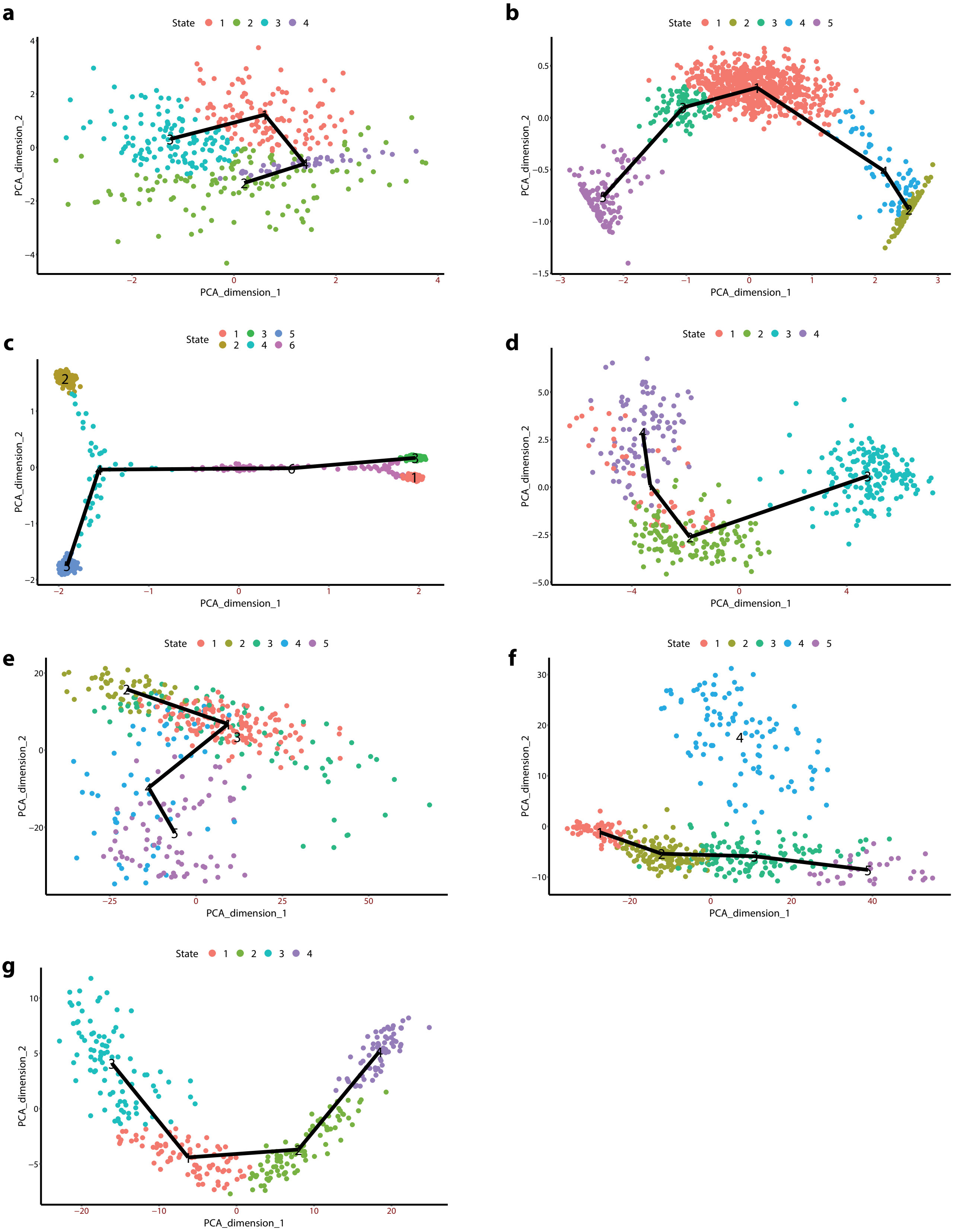

Figure C: **TSCAN results of seven datasets. (a-g)** are results for Simu1, Simu2, Simu3, Guo's dataset, Nef's dataset, Xu's dataset, and Furlan's dataset, respectively. As TSCAN conducts a scaling of the gene expression matrix in its algorithm, the PCA plots differ from other methods. The different colors represent different groups. The black line is the minimum spanning tree between clusters.

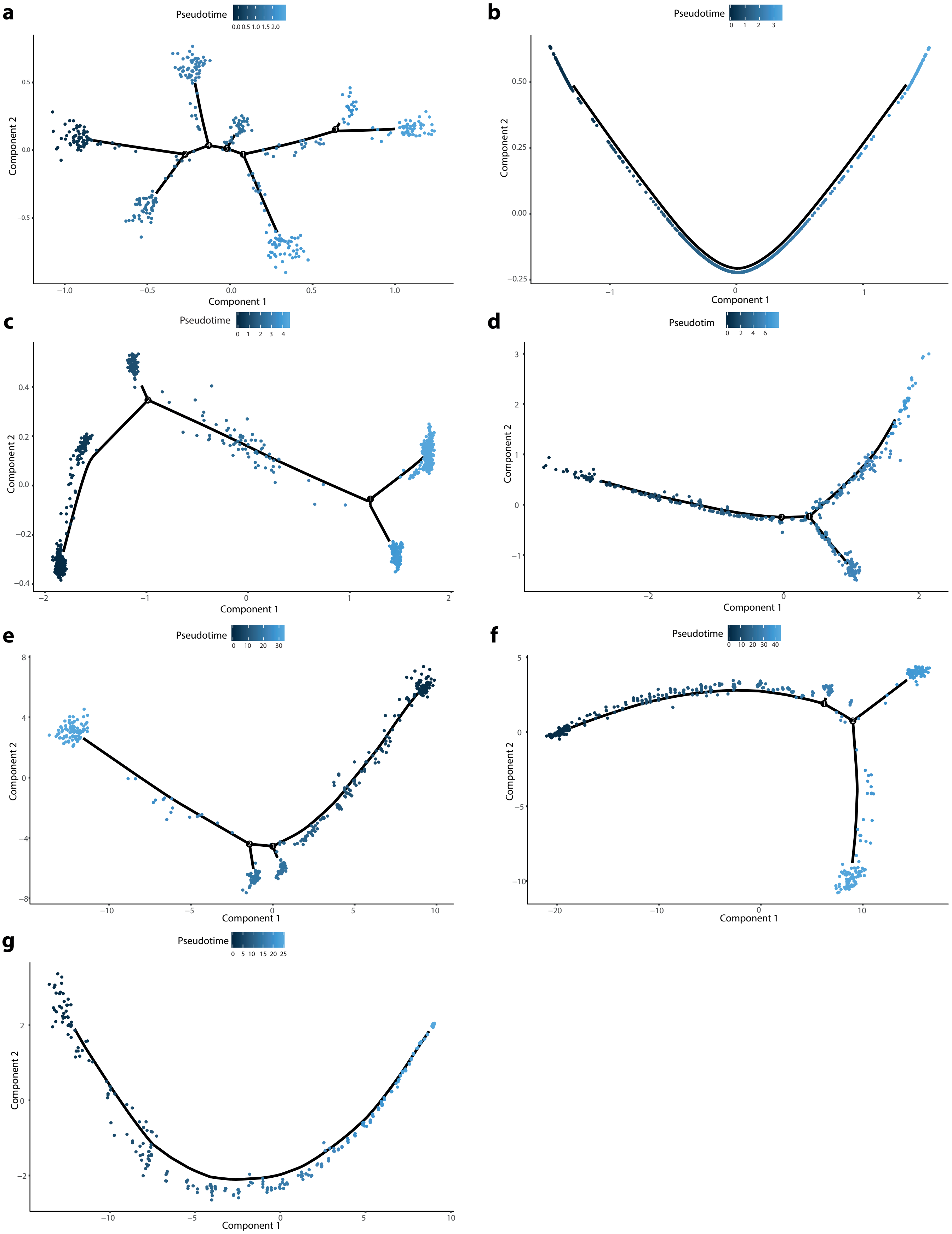

Figure D: **Monocle2 results of seven datasets.** **(a-g)** are results for Simu1, Simu2, Simu3, Guo's dataset, Nef's dataset, Xu's dataset, and Furlan's dataset, respectively. The differentiation tree structure is plotted by the black line, and the pseudo-time is shown by the color.

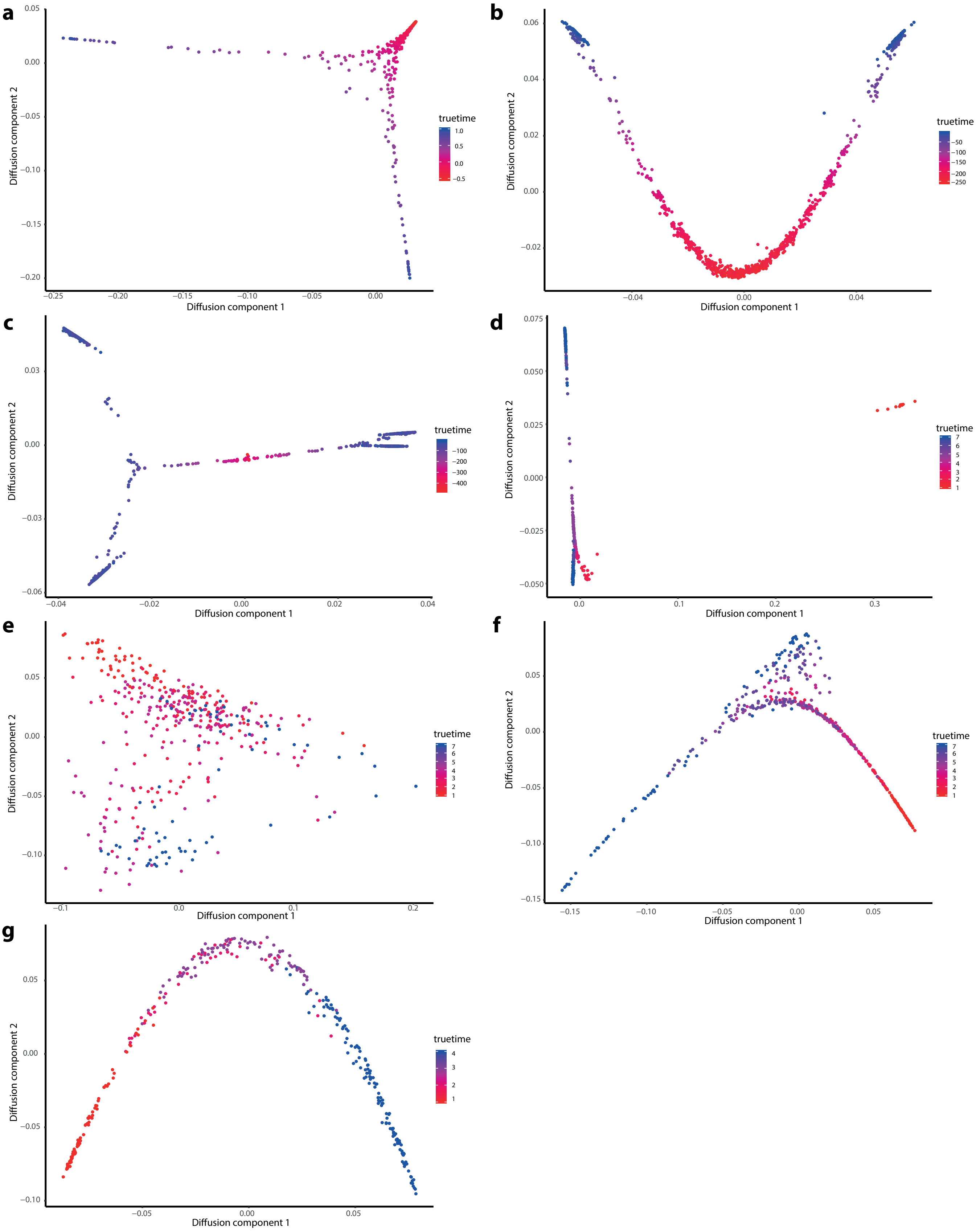

Figure E: **Diffusion Map results of seven datasets.** **(a-g)** are results for Simu1, Simu2, Simu3, Guo's dataset, Nef's dataset, Xu's dataset, and Furlan's dataset, respectively. The color represents the true time label. The pseudo-time is set as the first component of the diffusion map coordinates.

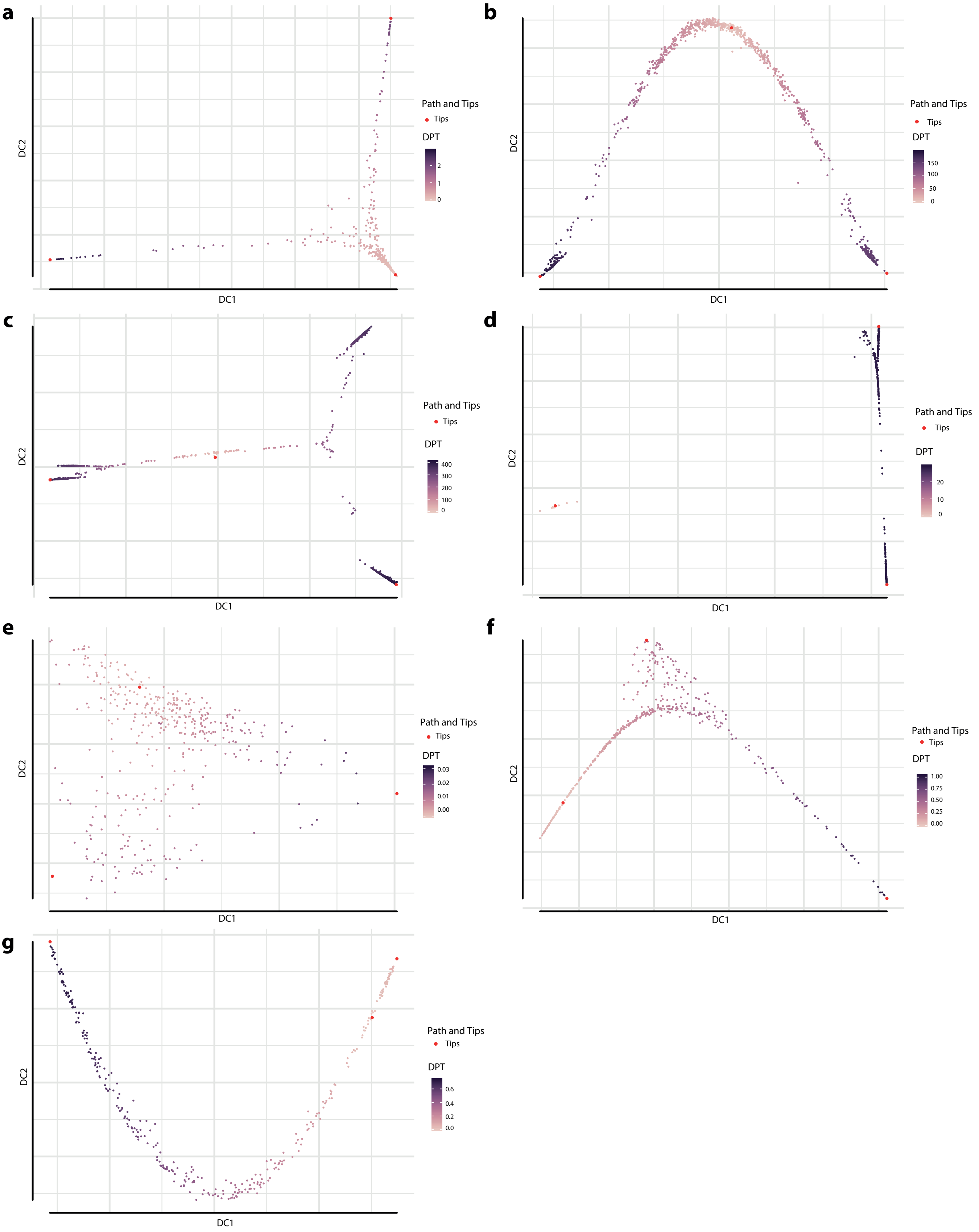

Figure F: **DPT results of seven datasets. (a-g)** are results for Simu1, Simu2, Simu3, Guo's dataset, Nef's dataset, Xu's dataset, and Furlan's dataset, respectively. The DPT pseudo-time is shown by the color.

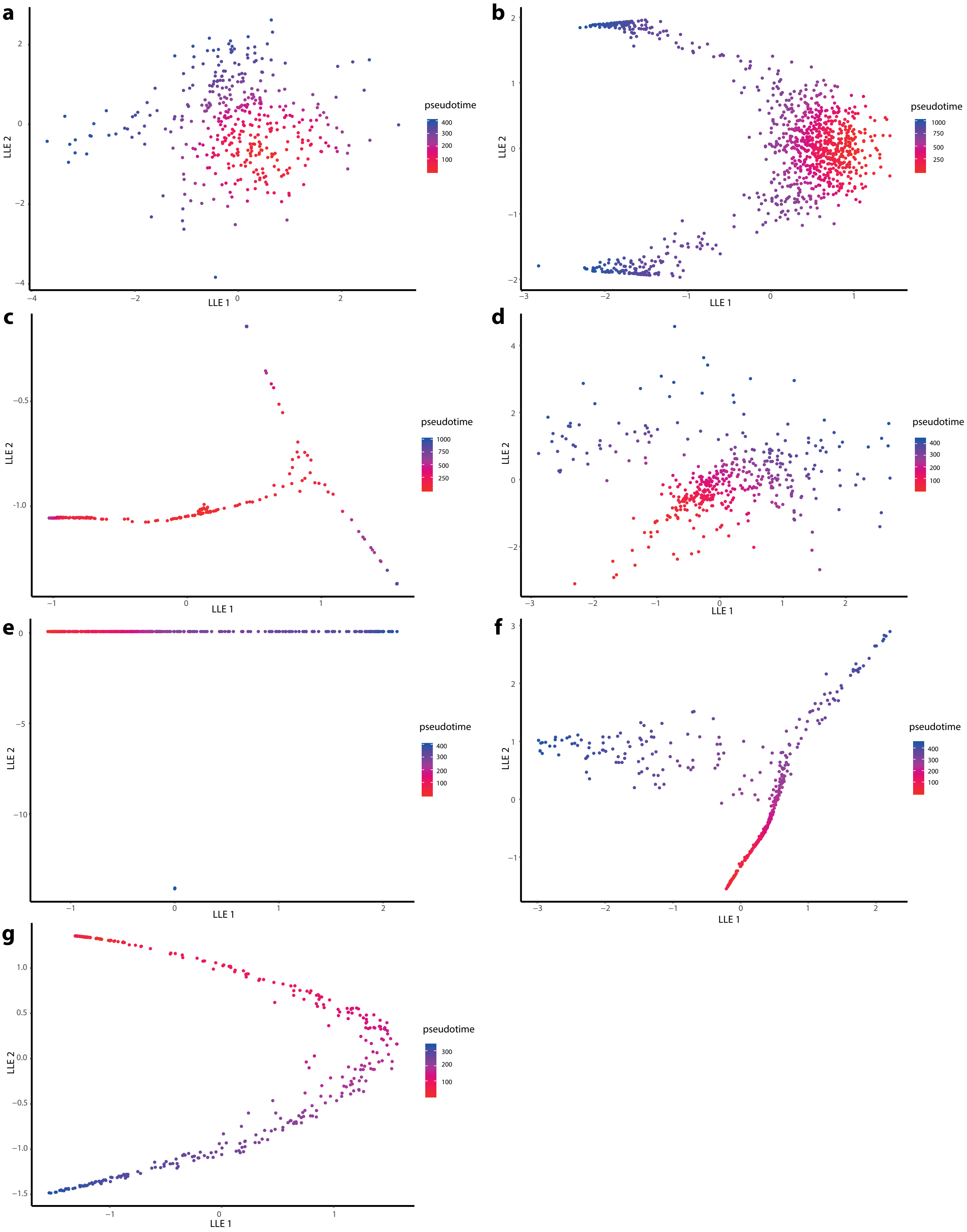

Figure G: **SLICER results of seven datasets. (a-g)** are results for Simu1, Simu2, Simu3, Guo's dataset, Nef's dataset, Xu's dataset, and Furlan's dataset, respectively. Local linear embedding is used for the dimension reduction. The pseudo-time is shown by the color.

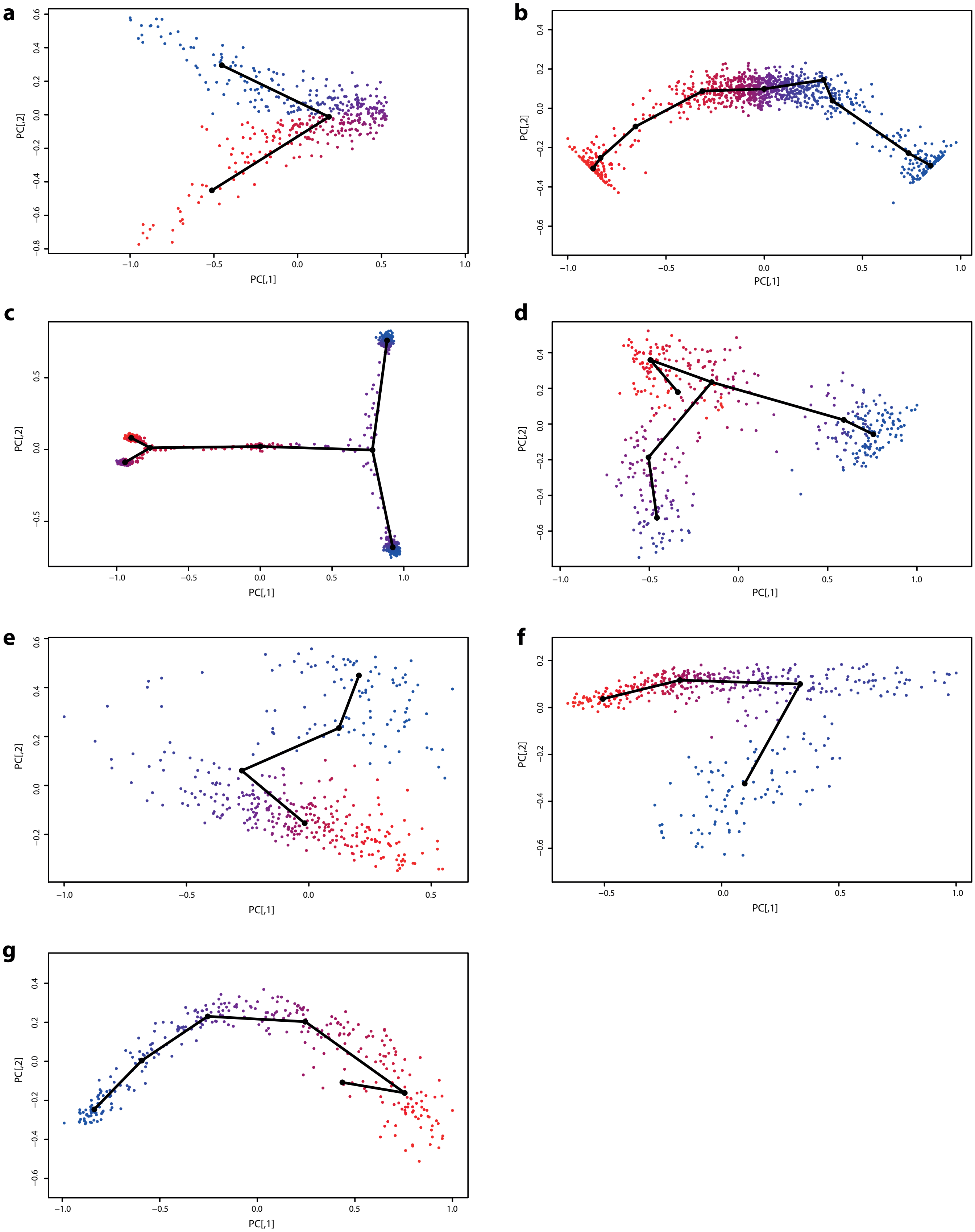

Figure H: **Slingshot results of seven datasets. (a-g)** are results for Simu1, Simu2, Simu3, Guo's dataset, Nef's dataset, Xu's dataset, and Furlan's dataset, respectively. The black line is the tree structure between clusters. The pseudo-time is shown by the color. The cells develop from red (high potential, small pseudo-time) to blue (low potential, large pseudo-time).

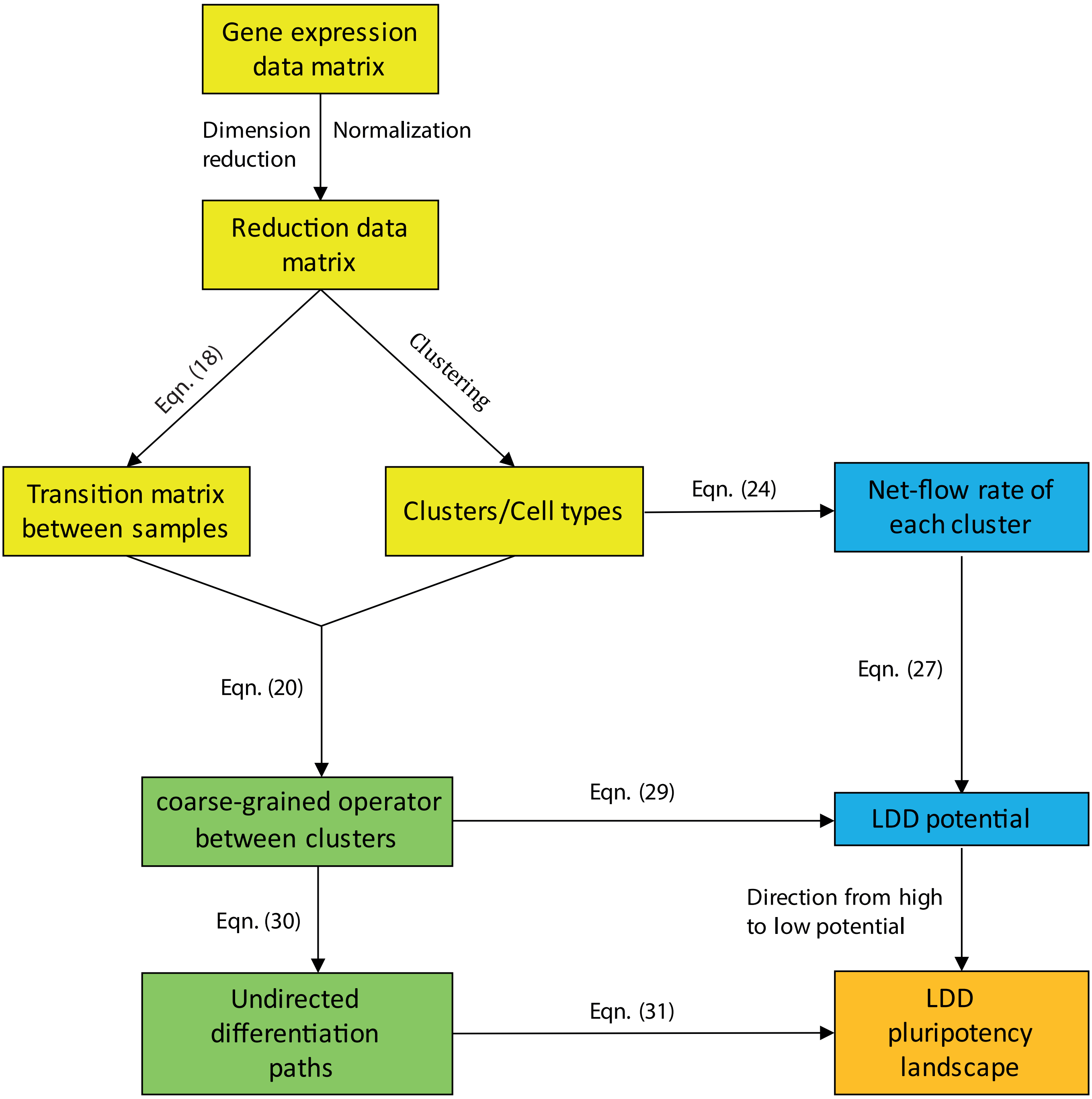

Figure I: **Detailed flowchart of the LDD procedure.** The equations noted in the flowchart are the corresponding equations in the main text.

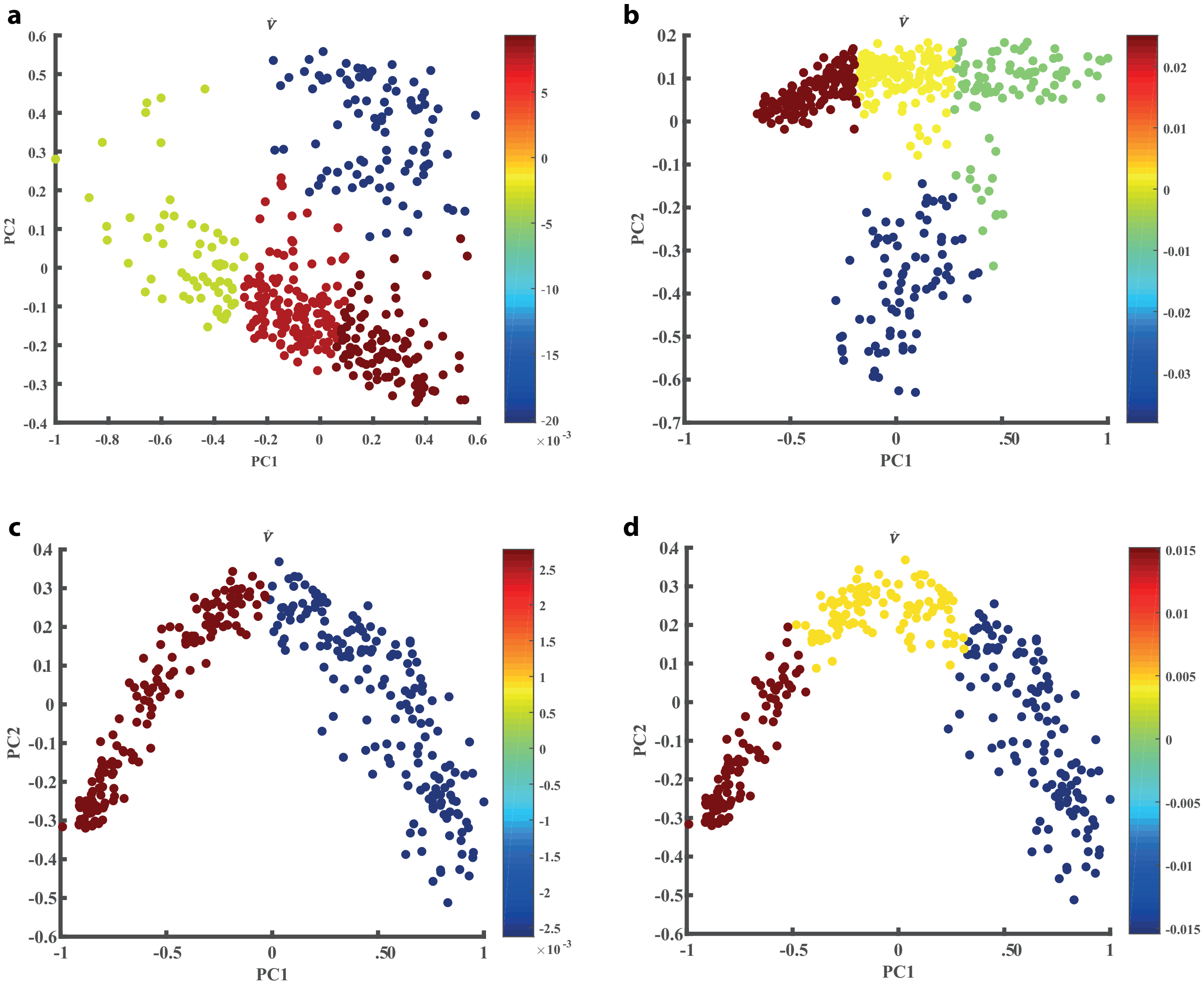

Figure J: **LDD results under different cluster numbers.** **(a)** Nef's dataset with four clusters. **(b)** Xu's dataset with four clusters. **(c)** Furlan's dataset with two clusters. **(d)** Furlan's dataset with three clusters.

[1] Philipp Angerer, Laleh Haghverdi, Maren Büttner, Fabian J Theis, Carsten Marr, and Florian Buettner. destiny: diffusion maps for large-scale single-cell data in R. *Bioinformatics*, 32(8):1241–1243, 2015.

[2] Alessandro Furlan, Vyacheslav Dyachuk, Maria Eleni Kastriti, Laura Calvo-Enrique, Hind Abdo, Saida Hadjab, Tatiana Chontorotzea, Natalia Akkuratova, Dmitry Usoskin, Dmitry Kamenev, et al. Multipotent peripheral glial cells generate neuroendocrine cells of the adrenal medulla. *Science*, 357(6346):eaal3753, 2017.

[3] Guoji Guo, Mikael Huss, Guo Qing Tong, Chaoyang Wang, Li Li Sun, Neil D Clarke, and Paul Robson. Resolution of cell fate decisions revealed by single-cell gene expression analysis from zygote to blastocyst. *Dev. Cell*, 18(4):675–685, 2010.

[4] Laleh Haghverdi, Florian Buettner, and Fabian J Theis. Diffusion maps for high-dimensional single-cell analysis of differentiation data. *Bioinformatics*, 31(18):2989–2998, 2015.

[5] Laleh Haghverdi, Maren Buettner, F Alexander Wolf, Florian Buettner, and Fabian J Theis. Diffusion pseudotime robustly reconstructs lineage branching. *Nat. Methods*, 13(10):845–848, 2016.

[6] Zhicheng Ji and Hongkai Ji. TSCAN: Pseudo-time reconstruction and evaluation in single-cell RNA-seq analysis. *Nucleic Acids Res.*, 44(13):e117, 2016.

[7] Xiaojie Qiu, Qi Mao, Ying Tang, Li Wang, Raghav Chawla, Hannah A Pliner, and Cole Trapnell. Reversed graph embedding resolves complex single-cell trajectories. *Nat. Methods*, 14(10):979–982, 2017.

[8] Isabelle Stévant, Yasmine Neirijnck, Christelle Borel, Jessica Escoffier, Lee B Smith, Stylianos E Antonarakis, Emmanouil T Dermitzakis, and Serge Nef. Deciphering cell lineage specification during male sex determination with single-cell RNA sequencing. *Cell Rep.*, 22(6):1589–1599, 2018.

[9] Kelly Street, Davide Risso, Russell B Fletcher, Diya Das, John Ngai, Nir Yosef, Elizabeth Purdom, and Sandrine Dudoit. Slingshot: Cell lineage and pseudotime inference for single-cell transcriptomics. *BMC genomics*, 19(1):477, 2018.

[10] Joshua D Welch, Alexander J Hartemink, and Jan F Prins. SLICER: inferring branched, nonlinear cellular trajectories from single cell RNA-seq data. *Genome Biol.*, 17(1):106, 2016.

[11] Li Yang, Wei-Hua Wang, Wei-Lin Qiu, Zhen Guo, Erfei Bi, and Cheng-Ran Xu. A single-cell transcriptomic analysis reveals precise pathways and regulatory mechanisms underlying hepatoblast differentiation. *Hepatology*, 66(5):1387–1401, 2017.
